# Methamphetamine Accelerates Cellular Senescence through Stimulation of *De Novo* Ceramide Biosynthesis

**DOI:** 10.1371/journal.pone.0116961

**Published:** 2015-02-11

**Authors:** Giuseppe Astarita, Agnesa Avanesian, Benedetto Grimaldi, Natalia Realini, Zuzana Justinova, Leight V. Panlilio, Abdul Basit, Steven R. Goldberg, Daniele Piomelli

**Affiliations:** 1 Department of Pharmacology, University of California Irvine, Irvine, California, United States of America; 2 Drug Discovery and Development, Italian Institute of Technology, Genoa, Italy; 3 Preclinical Pharmacology Section, Behavioral Neuroscience Research Branch, National Institute on Drug Abuse, Baltimore, Maryland, United States of America; 4 Departement of Psychiatry, MPRC, University of Maryland School of Medicine, Baltimore, Maryland, United States of America; 5 Department of Anatomy and Neurobiology, University of California Irvine, Irvine, California, United States of America; University G. D’Annunzio, ITALY

## Abstract

Methamphetamine is a highly addictive psychostimulant that causes profound damage to the brain and other body organs. Post mortem studies of human tissues have linked the use of this drug to diseases associated with aging, such as coronary atherosclerosis and pulmonary fibrosis, but the molecular mechanism underlying these findings remains unknown. Here we used functional lipidomics and transcriptomics experiments to study abnormalities in lipid metabolism in select regions of the brain and, to a greater extent, peripheral organs and tissues of rats that self-administered methamphetamine. Experiments in various cellular models (primary mouse fibroblasts and myotubes) allowed us to investigate the molecular mechanisms of systemic inflammation and cellular aging related to methamphetamine abuse. We report now that methamphetamine accelerates cellular senescence and activates transcription of genes involved in cell-cycle control and inflammation by stimulating production of the sphingolipid messenger ceramide. This pathogenic cascade is triggered by reactive oxygen species, likely generated through methamphetamine metabolism *via* cytochrome P_450_, and involves the recruitment of nuclear factor-κB (NF-κB) to induce expression of enzymes in the *de novo* pathway of ceramide biosynthesis. Inhibitors of NF-κB signaling and ceramide formation prevent methamphetamine-induced senescence and systemic inflammation in rats self-administering the drug, attenuating their health deterioration. The results suggest new therapeutic strategies to reduce the adverse consequences of methamphetamine abuse and improve effectiveness of abstinence treatments.

## Introduction

The abuse of methamphetamine (D-meth) is a major health concern in industrialized countries, where a socially diverse group of users seeks the drug for its desirable psychological and physiological effects—a combination of euphoria, heightened arousal, reduced appetite and decreased fatigue [1]. Due to its highly addictive properties, D-meth also initiates an escalation in frequency and intensity of use, which brings about a host of negative symptoms such as panic and psychosis [1]. The pharmacological mechanism underlying these diverse actions is well understood—D-meth acts in the central nervous system to interrupt the reuptake of dopamine and other amine neurotransmitters, and facilitate their release into the synaptic space [1]. With prolonged drug exposure, these neurochemical alterations can lead to long-lasting damage to the brain, especially in structures containing dopaminergic axon terminals, which contribute to the emotional and cognitive problems experienced by D-meth addicts [2,3,4]. This transition to pathology has been attributed to a series of concurring events that include disruption of neuronal redox homeostasis [5,6,7,8], activation of apoptotic and necrotic processes [9], and recruitment of pro-inflammatory pathways dependent on the transcription nuclear factor NF-κB [7,10,11,12].

In addition to being neurotoxic, D-meth exerts widespread harmful effects throughout the body. Perhaps most striking among them is the extreme tooth decay (‘meth mouth’) that exacerbates the prematurely aged physical appearance typical of D-meth addicts [13]. While several abused drugs may increase the speed of organismal decline [14,15], *post mortem* studies have specifically linked the protracted use of D-meth to various pathologies characteristic of old age, including coronary artery atherosclerosis, pulmonary fibrosis and liver steatosis [16,17,18]. Nevertheless, the molecular mechanism through which D-meth might accelerate the emergence of age-related diseases remains unknown. Here we report that D-meth promotes cellular senescence and activates transcription of genes involved in inflammation and aging through a mechanism that requires increased biosynthesis of the sphingolipid messenger ceramide. These results shed new light on the molecular mechanism underlying D-meth toxicity and identify potential therapeutic targets to attenuate the life-threatening consequences of D-meth exposure in recovering addicts.

## Materials and Methods

### Chemicals

D-Methamphetamine (D-meth), L-methamphetamine (L-meth), L-cycloserine, myriocin, thalidomide, cimetidine, quinidine, SKF-525A and clotrimazole were purchased from Sigma Aldrich (St. Louis, Missouri). Fumonisin B_1_ (FB1), C6 and C8 ceramide, and HET-0016 were from Cayman Chemicals (Ann Arbor, Michigan). 5′-aminosalicylic acid and JSH-23 were from Santa Cruz Biotechnology (Santa Crux, CA).

### Animals

Adult male C57BL/6 mice (25–30 g; Charles River, Wilmington, MA) and Sprague-Dawley rats (360–440 g; Charles River) were kept in a temperature-controlled environment with a 12 h light/12 h dark cycle, with standard chow and water ad libitum. Rats were individually housed. All procedures met the National Institutes of Health guidelines for the care and use of laboratory animals, as well as the Guidelines for the Care and Use of Mammals in Neuroscience and Behavioral Research (National Research Council 2003), and were approved by the Institutional Animal Care and Use Committees of the University of California, Irvine, and the Intramural Research Program of the National Institute on Drug Abuse (NIDA).

### Methamphetamine self-administration

Surgery

Silastic intravenous catheters were implanted into the external jugular vein under anesthesia with a mixture of ketamine and xylazine (60 and 10 mg-kg^-1^, i.p., respectively). Catheters exited the skin behind the ear. After catheter implantation, a nylon bolt glued to an acrylic mesh was implanted subcutaneously in the midscapular region. The nylon bolt served as a tether, preventing the catheter from being pulled out during self-administration sessions. Following surgery, the catheters were flushed daily during the first week with 0.2–0.3 ml of a solution containing cephalosporin (100 mg-ml^-1^; cefazolin USP; Hospira Inc., Lake Forest, IL, USA) and flushed with saline before and after each daily session to maintain patency.

Apparatus

We used 18 standard operant-conditioning chambers (Coulbourn Instruments, Lehigh Valley, PA), which contained a white house light and two holes with nose-poke operanda on either side of a food trough. Upon activation, each nose poke produced a brief feedback tone. One hole was defined as active (left in 9 chambers, right in the remaining 9) and the other hole as inactive. D-meth or saline were delivered through Tygon tubing, protected by a metal spring and suspended through the ceiling of the experimental chamber from a single-channel fluid swivel. The tubing was attached to a syringe pump (Harvard Apparatus, South Natick, MA), which was programmed to deliver 2 s injections. The injected volume was adjusted for every animal to deliver a D-meth dose of 0.1 mg-kg^-1^ per injection. Experimental events were controlled by microcomputers using MED Associates interfaces and software (Med Associates Inc., East Fairfield, VT).

Procedure

Each experimental group of rats was divided into 2 subgroups, which were tested at the same time. Subgroup 2 served as yoked control and passively received a saline injection each time a response-contingent injection of D-meth was self-administered by a Subgroup 1 rat. Nose-poke responses by yoked control rats were recorded, but had no programmed consequences. Eight consecutive 15 h sessions were always conducted between 4 pm and 8 am. At the start of each session, a white light was turned on and a priming injection of D-meth (0.1 mg-kg^-1^) or saline sufficient to fill the “dead” space of the catheter, was automatically delivered. Rats learned to self-administer D-meth under one-response, fixed ratio schedule (FR1). Each nose-poke response in the active hole (FR1) produced a delivery of D-meth injection (0.1 mg-kg^-1^) followed by a 30 s timeout, during which the chamber was dark and responses in either hole had no programmed consequences. Responses in the inactive hole were recorded, but had no programmed consequences.

Tissue collection

The rats were killed by decapitation 2 h after the end of the eighth session. Brain, liver, heart, kidney (left), spleen, pancreas, testis, epididymal fat, skeletal muscle (vastus lateralis), and skin (hind paw) were harvested from each rat. The tissues were rinsed in a mix of RNase-free water with DEPC-treated PBS and blotted with sterile gauze. Brains and livers were snap-frozen in isopentane. All other tissues were snap-frozen in liquid nitrogen. Tissues were wrapped in aluminum foil and stored in -80°C.

### Acute methamphetamine administration

Three groups of male Sprague-Dawley rats were used. Group 1 received two injections of D-meth (10 mg-kg^-1^, intraperitoneal, i.p., n = 6) or saline (n = 6), one every 2 h. Group 2 received two injections of saline (n = 6) or D-meth (1.5 mg-kg^-1^, i.p., n = 6), one every 2 h. Group 3 received two i.p. injections of saline (n = 6) or D-meth (1.5 mg-kg^-1^, n = 5; 5 mg-kg^-1^, n = 5; 10 mg-kg^-1^, n = 6), one every 2 h. Animals were killed by decapitation 2 h after the last injection. Tissues were harvested, snap-frozen in liquid nitrogen and stored at -80°C for analyses.

### Cell cultures

Immortalized mouse embryonic fibroblasts (MEF) were purchased from American Type Culture Collection (Manassas, VA), murine C2C12 cells were a kind gift of Dr. Maria Pennuto’s group (Italian Institute of Technology, Genova, Italy), primary MEF cultures were prepared from C57BL/6 mouse embryos, as described [19]. See Supplemental information for detailed culture methods.

### Lipid extractions and analysis

Lipid extractions were carried out as described [20]. A detailed description of the extraction procedure and LC/MS conditions used for lipid analysis is reported in Supporting Material and Methods.

### D-Methamphetamine measurements

A detailed description of the extraction procedure and LC/MS conditions used for D-Methamphetamine measurements is reported in Supporting Material and Methods.

### Ceramide synthase activity

Ceramide synthase activity was measured as described [21]. A detailed description of assay conditions is reported in the Supporting Material and Methods.

### Senescence and cell toxicity assays

*Senescence-associated β-Gal staining* was performed as previously reported [22]. DNA replication assay, Crystal Violet Staining, Population Doublings counting, MTT (3-(4,5-dimethylthiazol-2-yl)-2,5-diphenyltetrazolium bromide) and LDH (lactate dehydrogenase) assays were used to measure cell viability and are fully described in the Supporting Material and Methods.

### Gene expression and silencing

See S1 Materials and Methods and S8 Table for detailed method.

### Reactive Oxygen Species (ROS) Production

ROS production was measured using the fluorescent probe CM-H2DCFDA (Invitrogen). MEFs were grown in Dulbecco’s Modified Eagle’s Medium (DMEM) without phenol red. Cells were plated to subconfluence in 12-well plates, washed 3 times with pre-warmed Phosphate Buffered Saline (PBS) and loaded for 30 min at 37°C/5% CO_2_ with 5 mM CM-H2DCFDA in DMEM without phenol red (loading medium). The loading medium was removed and pre-warmed fresh medium containing the different cytochromes P450 (CYP) inhibitors in presence or absence of D-meth was added. Fluorescence (excitation at 485 nm, emission at 530 nm) was analyzed immediately, then cells were incubated at 37°C and 5% CO_2_, and fluorescence was measured at the indicated time points. ROS rate versus control (%) was calculated subtracting the percentage of ROS increasing from time zero in the D-meth-treated samples to the percentage of ROS increasing from time zero in the vehicle treated samples.

### Chromatin immunoprecipitation (ChIP)

We used the two-step cross-linking method described in Nowak et al. [23]. See Supporting Material and Methods for detailed description.

### Statistical analyses

See S1 Materials and Methods for details.

## Results

### Methamphetamine self-administration increases tissue ceramide levels

**Fig 1:**
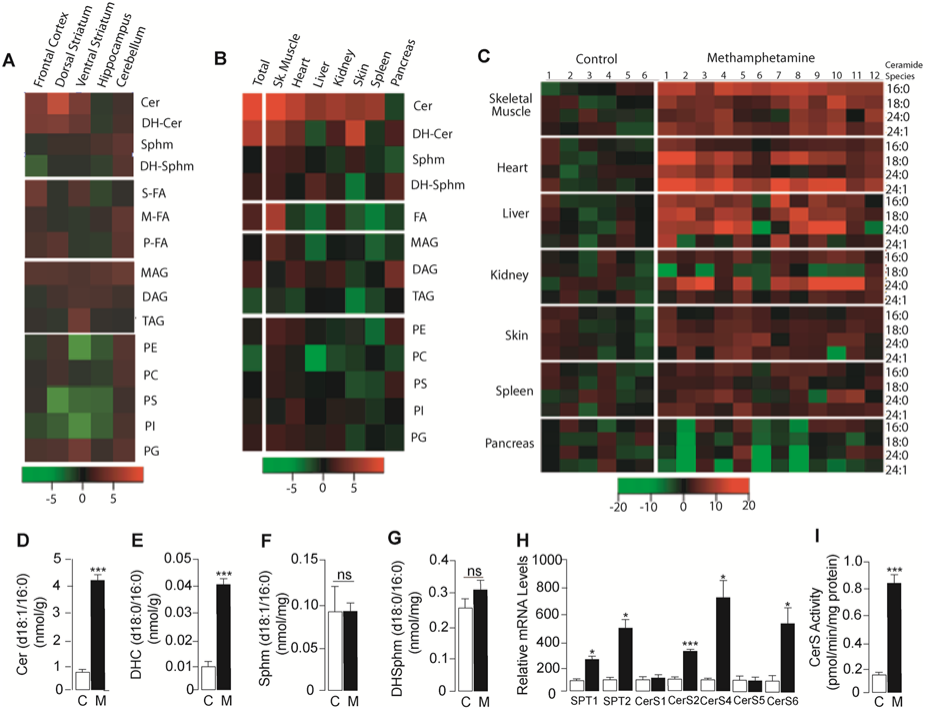
Lipidome-wide profiles in various tissues of rats self-administering D-meth. (A, B) Heat-maps showing changes in the levels of lipid classes (rows) in (A) brain regions and (A) peripheral organs of rats that self-administered D-meth for 8 days, compared to control rats receiving yoked saline injections. (C) Heat-map showing changes in the levels of various ceramide species (rows) in peripheral tissues of rats self-administering D-meth relative to control rats; columns show data from individual animals. Heat maps were generated by normalizing the data for each lipid species relative to the mean and standard error of the control group, such that the color of each subject’s cell indicates the number of standard errors above (red cells) or below (green cells) the mean of the control group. (D-I) Levels of (D) ceramide, (E) dihydroceramide, (F) sphingomyelin, (G) dihydrosphingomyelin, (H) mRNAs encoding for enzymes of *de novo* ceramide biosynthesis in skeletal muscle (vastus lateralis), and (I) ceramide synthase activity in skeletal muscle; control (C), open bars; rats self-administering D-meth (M), filled bars. Cer, ceramide; DAG, diacylglycerol; DH-Cer, dihydroceramide; DH-Sphm, dihydrosphingomyelin; MAG, monoacylglycerol; M-FA, monounsaturated fatty acid; PC, phosphatidylcholine; PE, phosphatidylethanolamine; P-FA, polyunsaturated fatty acid; PG, phosphatidylglycerol; PI, phosphatidylinositol; PS, phosphatidylserine; S-FA, saturated fatty acid; Sphm, sphingomyelin; TAG, triacylglycerol. Values are expressed as mean±s.e.m. *P<0.05, ***P<0.001, n.s., non significant; two-tailed Student’s *t* test (n = 6–12).

To determine whether abnormalities in lipid metabolism might contribute to the harmful effects of D-meth, we conducted an unbiased lipidomic survey in organs and tissues of rats that self-administered the drug intravenously [24]. A large percentage of animals taking D-meth escalated to high exposure levels over a few days (S1 Fig.), a behavior that closely mimics abuse of the drug in humans [24]. After 8 days of access to D-meth, the rats were euthanized and tissues were subjected to solvent extraction. The extracts were fractionated by liquid chromatography (LC) and representative lipid class constituents were identified and quantified by mass spectrometry (MS). The data were initially processed using heat-maps to facilitate visual inspection of broad regions of interest in the lipidome (Fig. 1A-C). Subsequent quantitative analyses were conducted separately using appropriate internal standards (Fig. 1D-G, S1–S4 Tables).

The most striking difference between rats self-administering D-meth and those receiving yoked injections of vehicle was an increase in ceramide content in select regions of the brain (Fig. 1A, S1 and S2 Tables) and, to a greater extent, peripheral organs and tissues (Fig. 1B, S3 and S4 Tables). The effect was prominent in skeletal muscle (Fig. 1C-D), and involved multiple ceramide species—including (d18:1/16:0), (d18:1/18:0), (d18:1/24:0) and [d18:1/24:1(15Z)] (Fig. 1C). The levels of dihydroceramide (d18:0/16:0), an obligatory biosynthetic precursor for ceramide (d18:1/16:0) through the *de novo* pathway [25], were also higher in rats that self-administered D-meth than in yoked controls (Fig. 1E). By contrast, there was no detectable difference in the levels of sphingomyelin (d18:1/16:0) or dihydrosphingomyelin (d18:0/16:0) (Fig. 1F-G, S3 Table), which generate ceramide through sphingomyelinase-mediated hydrolysis [25]. Consistent with an involvement of *de novo* ceramide biosynthesis, D-meth self-administration was associated with accrued transcription of ceramide-synthesizing enzymes—including serine palmitoyltransferases (SPT1 and SPT2) and ceramide synthases (CerS2, 4 and 6; Fig. 1H, S5 and S6 Tables)—and heightened tissue CerS activity (Fig. 1I). The results suggest that the production of ceramides—a family of sphingolipid messengers involved in senescence, inflammation and apoptosis [26,27,28]—is abnormally elevated in tissues of rats that self-administer D-meth.

### Methamphetamine stimulates de novo ceramide biosynthesis

We next examined whether acute exposure to D-meth might exert similar effects to those seen with D-meth self-administration. We used a non-contingent drug regimen involving two injections of D-meth separated by a 2 h interval, widely utilized to mimic the ‘runs’ wherein abusers binge on the drug [29]. When administered at a cumulative dose of 20 mg-kg^-1^, D-meth increased ceramide content, CerS6 transcription and CerS activity in rat skeletal muscle (S2A–C Fig.). A lower cumulative drug dosage (10 mg-kg^-1^ in two injections of 5 mg-kg^-1^ each) increased CerS6 transcription (S2B Fig.) and produced a trend toward increased ceramide synthase activity (S2C Fig.), but had no statistically detectable effect on ceramide levels (S2A Fig.).

**Fig 2:**
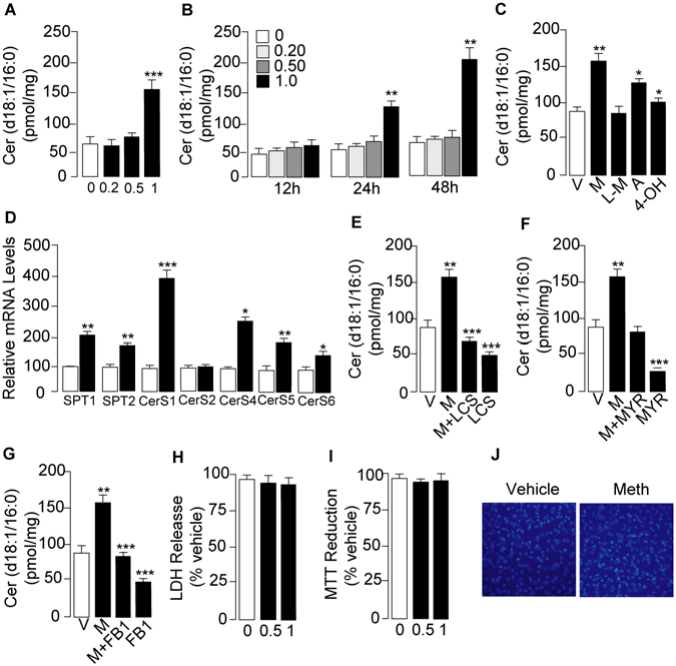
D-meth stimulates ceramide production in primary MEF cultures. (A) Concentration- dependence and (B) time-course of the effects of D-meth on ceramide levels. D-meth concentrations are expressed in mM. (C) Effects of various phenethylamines on ceramide levels; D-meth (M, 1 mM) L-meth (LM, 1 mM), D-amphetamine (A, 1mM), and 4-hydroxy-D-methamphetamine (4-OH, 1 mM). (D) Effects of D-meth (1 mM, 24 h) on transcription of enzymes in *de novo* ceramide biosynthesis. SPT, serine palmitoyl-Coenzyme A transferase; CerS, ceramide synthase. (E-G) Effects of the SPT inhibitors (E) L-cycloserine (L-CS, 30 μM), (F) myriocin (MYR, 10 μM), and (G) the CerS inhibitor fumonisin B_1_ (FB1, 50 μM) on ceramide levels in cells exposed to D-meth (M, 1 mM, 24 h) or vehicle. Cells were exposed to the drugs for 24 h. (H-J) Effects of D-meth (0–1 mM, 48 h) on MEF viability as assessed by (H) lactate dehydrogenase (LDH) release, (I) MTT ((3-(4,5-dimethylthiazol-2-yl)-2,5-diphenyltetrazolium bromide) reduction and (J) 4′,6-diamidino-2-phenylindole (DAPI) nuclear staining. Values are expressed as mean±s.e.m. of three separate experiments, each performed in triplicate. *P<0.05, **P<0.01, ***P<0.001, ANOVA followed by Bonferroni *post hoc* test.

To further characterize the mechanism through which D-meth stimulates ceramide production, and eliminate possible confounders due to indirect effects exerted by the drug *in vivo*, we used cultures of mouse embryonic fibroblasts (MEF) and differentiated C2C12 myoblasts. Incubation of primary MEF with D-meth (1mM) elicited a rise in ceramide content (Fig. 2A), which reached statistical significance 24h after addition of the drug (Fig. 2B). Similar results were obtained in an immortalized MEF line, where a detailed time-course analysis revealed that increases in ceramide levels were accompanied by parallel changes in the corresponding dihydroceramides, and were preceded by a rise in dihydrosphingosine (sphinganine), which both implicated *de novo* ceramide biosynthesis (S3 Fig.). Highlighting the selectivity of the effect of D-meth, two structurally related phenylethylamines, D-amphetamine and 4-hydroxy-D-methamphetamine, produced modest but significant changes in ceramide levels, while the enantiomer of D-meth, L-meth, was completely ineffective (Fig. 2C). The ability of D-meth to stimulate ceramide accumulation was confirmed using serum-differentiated C2C12 myoblasts. In these muscle-like cells, exposure to D-meth (0.1–1 mM) for 24 h caused concentration-dependent elevations in dihydrosphingosine, dihydroceramide (d18:0/16:0) and ceramide (d18:1/16:0), which were statistically significant at D-meth concentrations ≥0.5 mM (S4 Fig.).

In addition to increasing ceramide levels, in primary MEF cultures D-meth (1 mM, 24 h) also enhanced transcription of genes encoding for enzymes in *de novo* ceramide biosynthesis (Fig. 2D). Importantly, drugs that interrupt *de novo* ceramide production—including the SPT inhibitors, L-cycloserine and myriocin, and the CerS inhibitor, fumonisin B_1_ [30,31,32]—prevented D-meth-induced ceramide accumulation and decreased baseline ceramide formation (Fig. 2E-G). In these experiments, D-meth (1 mM, up to 48 h) caused no overt membrane damage (Fig. 2H) or mitochondrial dysfunction (Fig. 2I) and no change in cell viability (Fig. 2J), indicating that its effects on ceramide were not secondary to cell injury or cell death. As expected, under the same conditions cisplatin, a classic chemotherapy drug, produced substantial cell death (S5 Fig.). Collectively, the results outlined above suggest that D-meth stimulates *de novo* ceramide biosynthesis by interacting with a structurally selective molecular target. Because of its presence in cells that are not neurons, such as fibroblasts and differentiated myoblasts, this putative target is likely to be distinct from the neuronal monoamine transporters responsible for the stimulant properties of D-meth [1].

### Role of cytochrome P_450_ and NF-κB

**Fig 3:**
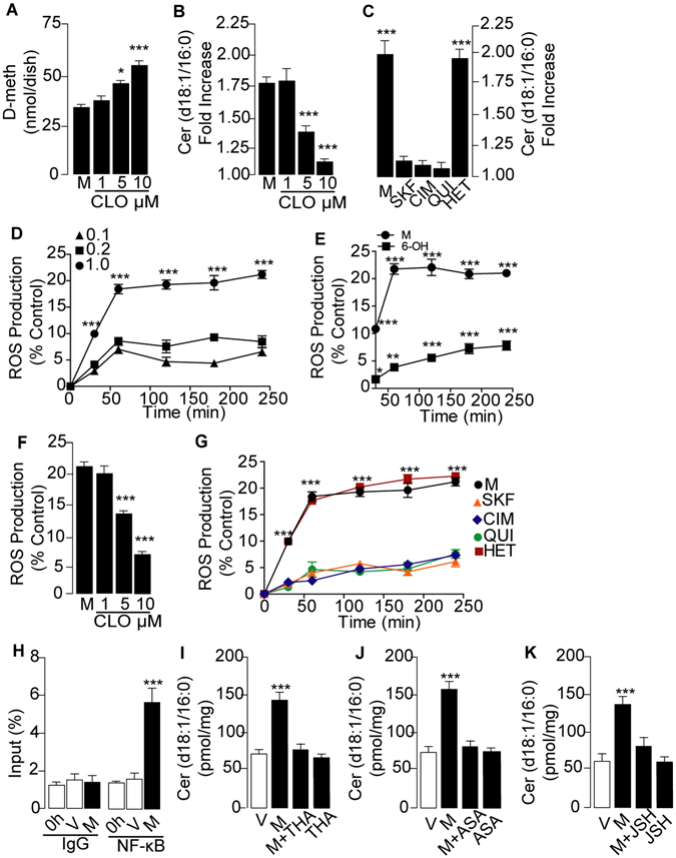
Role of cytochrome P_450_ (CYP) and nuclear factor (NF)- κB in D-meth-induced ceramide production. (A, B) Effects of pan-CYP inhibitor clotrimazole (CLO) on (A) cell-associated D-meth content and (B) ceramide levels. Primary MEF cultures were treated with D-meth (M, 1 mM) for 24 h and rinsed before extraction and quantification of D-meth by LC/MS. (C) Effects of CYP inhibitors on ceramide levels: SKF-525A (SKF, 10 μM), cimetidine (CIM, 10 μM), quinidine (QUI, 10 μM) and HET-0016 (HET, 10 μM). (D-E) Time-course of the effects of (D) D-meth (mM) and (E) 4-hydroxy-D-methamphetamine (4-OH, 1 mM) on ROS production. (F-G) Effects of (F) clotrimazole and (G) SKF-525A, cimetidine, quinidine and HET-0016 on ROS production. (H) Chromatin immunoprecipitation indicating recruitment of NF-κB to the TNF- promoter in primary MEF treated for 24 h with vehicle or D-meth (1 mM). Results are shown as percent of input (i.e., percent of the total amount of chromatin before immunoprecipitation). (I-K) Effects of NF-κB inhibitors (I) thalidomide (THA, 25 M), (J) 5-aminosalicylic acid (ASA, 10 μM) and (K) JSH-23 (JSH, 10 μM) on ceramide levels in MEF treated for 24h with vehicle or D-meth (1 mM). Values are expressed as mean±s.e.m. of three separate experiments, each performed in triplicate. *P<0.05, ***P<0.001, ANOVA followed by Bonferroni post hoc test.

D-meth is metabolized in humans and rodents by cytochrome P_450_ (CYP)-2D6, a widely distributed CYP isoform that catalyzes the oxidation of D-meth into D-amphetamine and 4-hydroxy-D-methamphetamine [33,34]. A by-product of this reaction is the formation of ROS [8], which are known to activate NF-κB-dependent stress-response signals that can lead to ceramide formation [35]. To determine whether CYP metabolism might be involved in D-meth-induced ceramide production, we blocked CYP activity in primary MEF cultures using a panel of five chemically distinct agents: clotrimazole, SKF-525A and cimetidine (three pan-CYP inhibitors), quinidine (selective for CYP2D6) and HET-0016 (selective for CYP4A). LC/MS analyses showed that incubation of MEF in the presence of clotrimazole (1–10 μM) increased the levels of non-metabolized D-meth (Fig. 3A) and concurrently decreased D-meth-induced ceramide accumulation (Fig. 3B), while exerting no effect on baseline ceramide content (in pmol-mg^-1^ protein, control: 52.9±5; clotrimazole 10 μM: 49.2±3; n = 3). Similarly to clotrimazole, SKF-525A, cimetidine and quinidine (each at 10 μM) normalized ceramide formation in D-meth-treated cells, whereas HET-0016 (10 μM) was ineffective (Fig. 3C).

As a further test of the role of ROS in D-meth-induced ceramide production, we determined whether exposure to D-meth stimulates ROS generation in MEF. As anticipated from previous studies [8], D-meth caused a concentration-dependent increase in ROS formation (Fig. 3D), whereas 4-hydroxy-D-methamphetamine (a product of D-meth metabolism via CYP2D6) [33,34] had little or no effect (Fig. 3E). The release of ROS evoked by D-meth was prevented by the pan-CYP inhibitors—clotrimazole (Fig. 3F), SKF-525A and cimetidine (Fig. 3G)—and the CYP2D6 inhibitor, quinidine (Fig. 3G), but not by the CYP4A inhibitor, HET-0016 (Fig. 3G).

An early cellular response to ROS formation is the recruitment of NF-κB [36,37], which can also be induced by D-meth [10,11]. Supporting a role for NF-κB activation in the effects of D-meth, we found that exposure of MEF cultures to the drug (1 mM, 24 h) resulted in a 4-fold increase in the amount of NF-κB p65 subunit bound to the TNF-α promoter (Fig. 3H). Moreover, pharmacological blockade of NF-κB using three different inhibitors—thalidomide (25 μM), 5-aminosalicylic acid (10 μM) and JSH-23 (10 μM)—prevented D-meth-induced ceramide accumulation (Fig. 3I-K) as well as D-meth-induced SPT and CerS transcription (S6 Fig.). Silencing expression of the p65 subunit of NF-κB with a selective short interfering (si) RNA (S7 Fig.) impaired the ability of D-meth (1 mM) to increase ceramide levels (S8 Fig.) and stimulate expression of enzymes of *de novo* ceramide biosynthesis (S9 Fig.). These findings are consistent with the possibility that CYP-mediated metabolism of D-meth induces formation of ROS and engages NF-κB, resulting in stimulation of ceramide biosynthesis.

### Methamphetamine accelerates cellular senescence

**Fig 4:**
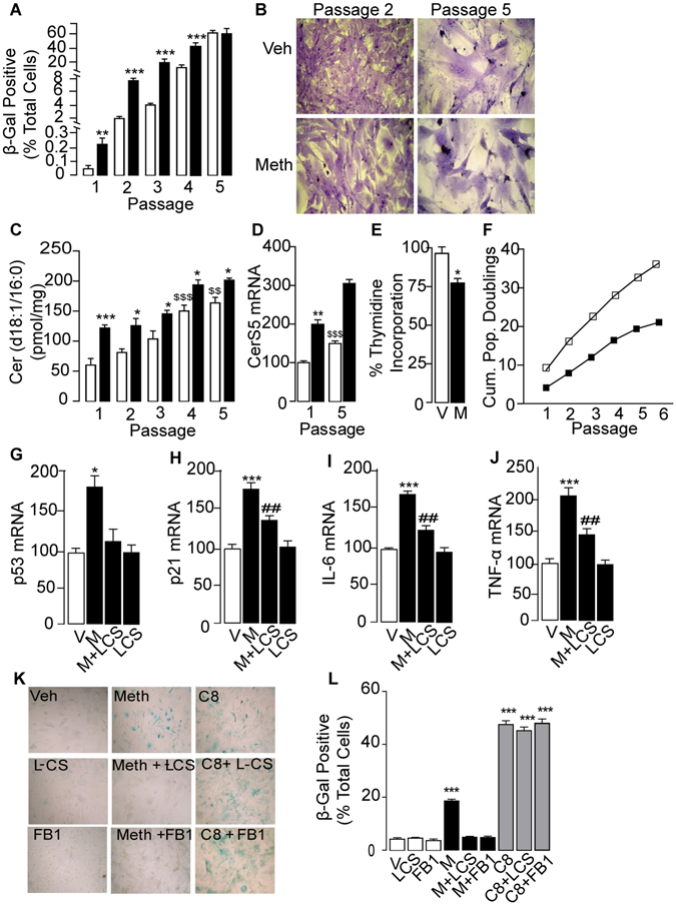
D-meth accelerates replicative senescence. (A-D) Effects of D-meth (1 mM, closed bars) or vehicle (open bars) on (A) senescence-associated -galactosidase (-Gal) staining, (B) cell morphology, (C) ceramide levels, and (D) expression of CerS5 mRNA in primary MEF cultures. (E-F) Effects of D-meth on replicative capacity assessed by (E) [^3^H]-thymidine incorporation into DNA and (F) number of cumulative population doublings (vehicle: open squares, D-meth: closed squares). (g-j) Effects of D-meth (M, 1 mM), L-cycloserine (L-CS, 30 μM) and combination of D-meth plus L-CS on transcription of senescence-associated markers: (G) p53, (H) p21, (I) IL-6 and (J) TNF-α. (K, L) Effects of L-CS (30 μM), fumonisin B_1_ (FB1, 50 μM) and cell-permeant C8 ceramide (10 μM) on -Gal expression elicited by D-meth (M, 1 mM). ANOVA followed by Bonferroni *post hoc* test: *P<0.05, **P<0.01, ***P<0.001, *vs*. vehicle; ^*$*^ *P*<0.05, ^*$$$*^ *P*<0.001, *vs* vehicle at passage 1. ^*##*^ *P*<0.01, *vs* D-meth.

A variety of stressful stimuli force proliferating cells into a state, called cellular senescence, characterized by cell-cycle arrest, apotosis resistance, and increased secretion of proinflammatory factors [38,39,40]. Since membrane-permeant ceramide analogs cause senescence in fibroblasts and other cell types [41,42,43], we asked whether D-meth-induced ceramide formation might exert a similar effect. For these experiments we used primary cultures of MEF, the senescence response of which has been most extensively characterized [41]. When cultured in control medium, primary MEF progressively developed a phenotype characterized by increased expression of senescence-associated β-galactosidase (β-Gal; Fig. 4A), flattened morphology (Fig. 4B), and higher levels of ceramide (Fig. 4C) and CerS5 mRNA (Fig. 4D). Culturing MEF in the presence of D-meth (1 mM, 48 h) intensified this senescent phenotype with decreased intensity as the cells approached the end of their proliferative capacity (Fig. 4A). The possibility that D-meth accelerates senescence, suggested by these observations, was further supported by results showing that treatment with D-meth reduced DNA synthesis (Fig. 4E), lowered the number of cell doublings (Fig. 4F), and stimulated the transcription of genes encoding for cell cycle-regulating proteins (p53, p21) and pro-inflammatory cytokines [interleukin-6 (IL-6) and tumor necrosis factor-α (TNF-α)] (Fig. 4G-J), which are all known to participate in senescence [38,39].

Next, to assess the contribution of ceramide to D-meth-induced senescence, we interrupted the *de novo* biosynthesis of this sphingolipid messenger using the SPT inhibitor, L-cycloserine (30 μM), or the CerS inhibitor, fumonisin B_1_ (50 μM). The compounds suppressed senescence-associated β-Gal expression in MEF treated with D-meth (1 mM, 48 h), but not in MEF treated with the cell-permeant ceramide analog C8 (10 μM, 48 h) (Fig. 4K-L). Additionally, L-cycloserine (30 μM) normalized transcription of p53, p21, IL-6 and TNF-α (Fig. 4G–J). The results suggest that D-meth accelerates cellular senescence in MEF through induction of *de novo* ceramide biosynthesis. This process appears to depend on the recruitment of NF-κB, as documented by the finding that thalidomide and siRNA silencing of NF-κB were each able to prevent it (S10 Fig.).

### Ceramide is a key effector of systemic metamphetamine toxicity

**Fig 5:**
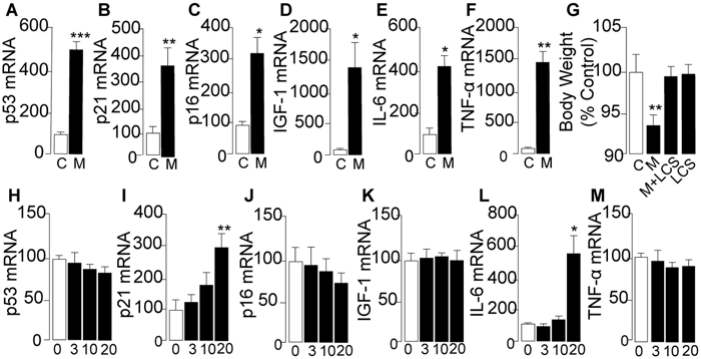
Enhanced transcription of senescence- and inflammation-associated markers in skeletal muscle of rats exposed to D-meth. (A–F) mRNA levels in skeletal muscle of control rats (C, open bars) and rats self-administering D-meth (M, closed bars): (A) p53; (B) p21; (C) p16; (D) IGF-1; (E) IL-6; (F) TNF-α. (G) Effects of D-meth self-administration (M), L-cycloserine (L-CS) treatment or a combination of D-meth and L-cycloserine (L-CS) on body weight. (H–M) mRNA levels in skeletal muscle of control rats and rats that received two 10 mg-kg^-1^ injections of D-meth in a 2-h period: (H) p53; (I) p21; (J) p16; (K) IGF-1; (L) IL-6; (M) TNF-α. Values are expressed as the mean±s.e.m. *P<0.05, **P<0.01, ***P<0.001, two-tailed Student’s *t* test. *P<0.05, **P<0.01, ***P<0.001, ANOVA followed by Bonferroni *post hoc* test.

The finding that D-meth promotes senescence in primary MEF cultures prompted us to ask whether self-administration of the drug might activate a transcription program suggestive of accelerated senescence *in vivo* [39]. Since many senescence-associated proteins are also involved in chronic inflammation [40], we reasoned that their abnormal expression might contribute to the systemic pathological state elicited by D-meth. As a first test of this idea, we quantified mRNA levels of various genetic markers of senescence and inflammation in rats self-administering D-meth. The transcription of six such markers—p53, p21, p16, IGF-1 [38,39,44]—was substantially increased in skeletal muscle after 8 days of voluntary D-meth intake, relative to yoked vehicle self-administration (Fig. 5A-F). Statistically significant elevations in TNF-α and IL-6 transcripts were also observed in heart and skin (S7 Table). These molecular changes were accompanied by a reduction in body weight (Fig. 5G), which is suggestive of sharp health deterioration. Acute non-contingent injections of D-meth, which closely mimic a drug binge (two 10 mg-kg^-1^ injections separated by a 2 h interval), increased transcription of p21 and IL-6 but had no effect on the levels of p53, p16, IGF-1 or TNF-α mRNA (Fig. 5H-M). This difference is noteworthy, because it suggests that prolonged intake of D-meth is necessary for the drug to fully engage molecular processes involved in senescence and inflammation.

**Fig 6:**
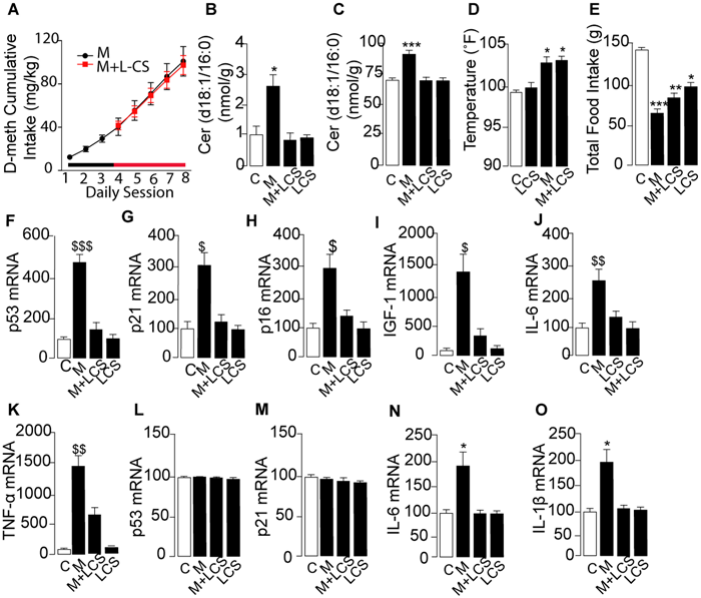
Effects of L-cycloserine (L-CS) on senescence- and inflammation-associated markers in skeletal muscle and brain of rats that self-administer D-meth. (A) Cumulative D-meth intake in rats receiving daily injections of vehicle (black circles) or L-CS (red squares). (B, C) Effects of D-meth self-administration, L-CS treatment or combination of D-meth plus L-CS on ceramide levels in (B) skeletal muscle and (C) brain dorsal striatum. (D–O) Effects of D-meth self-administration, L-CS treatment or combination of D-meth plus L-CS on (D) body temperature; (E) total food intake; (F–K) skeletal muscle mRNA levels of (F) p53; (G) p21; (H) p16; (i) IGF-1; (J) IL-6; (K) TNF-α; and (L-O) dorsal striatum mRNA levels of (L) p53; (M) p21; (N) IL-6; and (O) IL-1ß. Values are expressed as the mean±s.e.m. *P<0.05, **P<0.01, ***P<0.001, two-tailed Student’s *t* test. *P<0.05, **P<0.01, ***P<0.001, ANOVA followed by Bonferroni *post hoc* test.

Lastly, to evaluate the role of ceramide in systemic D-meth toxicity, we treated rats that self-administered D-meth with the SPT inhibitor L-cycloserine (10 mg-kg^-1^, twice daily for 2 days, followed by 20 mg-kg^-1^ twice daily for 3 days). The L-cycloserine regimen normalized ceramide content in skeletal muscle and brain tissue (dorsal striatum) of rats that self-administered D-meth, without changing baseline ceramide levels in animals that received yoked vehicle injections (Fig. 6B-C, respectively). L-cycloserine did not alter key centrally mediated actions of D-meth—including its ability to maintain self-administration (Fig. 6A), increase body temperature (Fig. 6D) and reduce food intake (Fig. 6E). Nevertheless, the SPT inhibitor corrected the abnormalities in body weight (Fig. 5G) and gene expression caused by D-meth in both skeletal muscle (Fig. 6F-K) and dorsal striatum (Fig. 6L-O), providing evidence that ceramide is an obligatory effector of the systemic inflammation and health deterioration produced by D-meth.

## Discussion

While conducting a survey of lipidomic abnormalities associated with self-administration of D-meth, we found that ceramide production is strongly increased in rats that voluntarily take the drug. This effect was clearly detectable in select regions of the brain, such as the dorsal striatum, but was particularly pronounced in peripheral organs known to undergo pathological changes following prolonged exposure to D-meth (e.g., skeletal muscle, heart and liver) [16,17,18]. In those organs, accrued ceramide biosynthesis was associated with activation of a senescence-like transcription program—characterized by enhanced expression of genes involved in cell-cycle control (e.g., p21, p16) and chronic inflammation (e.g., IL-6 and TNF-α)—which could be recapitulated *in vitro* by treating mouse fibroblasts or differentiated C2C12 myoblasts with D-meth. Importantly, pharmacological inhibitors of ceramide formation prevented the occurrence of these molecular events, and lowered D-meth-induced cellular and systemic toxicity. These studies suggest that modulation of ceramide signaling might be utilized therapeutically to attenuate the serious, often fatal health consequences of D-meth abuse and enhance the success of behavior-based abstinence programs.

Our lipidomic analyses show that D-meth self-administration in rats—a model that realistically captures abuse of the drug in humans [24]—is accompanied by changes in the levels of ceramide and its precursor dihydroceramide, which are suggestive of an up-regulation of the *de novo* pathway of ceramide biosynthesis. This possibility is supported by two additional observations: (*i*) D-meth increased transcription of genes encoding for two key enzyme families in that pathway, SPT and CerS; and (*ii*) pharmacological interference with the activities of those enzymes blocked D-meth-induced ceramide formation. How may D-meth influence SPT and CerS expression? Pharmacological evidence implicates CYP-mediated metabolism, which is responsible for the metabolic clearance of D-meth in humans and rodents [33,34]. Experiments with mouse fibroblasts show that (*i*) D-meth stimulates ROS generation, NF-κB activation and ceramide production; (*ii*) genetic or pharmacological interference with CYP or NF-κB activities prevents these effects; (*iii*) structural analogs of D-meth, such as L-meth and 4-hydroxy-D-methamphetamine, which are comparatively poor substrates for CYP-mediated oxidation, only partially mimic the effects of D-meth. Thus, an economical interpretation of the results outlined above is that D-meth metabolism *via* CYP generates ROS, which in turn activate SPT and CerS transcription through an NF-κB-dependent mechanism. This model is consistent with the greater vulnerability of human CYP2D6 extensive metabolizers to D-meth neurotoxicity [45], but does not exclude the possibility that D-meth might also stimulate ceramide biosynthesis through other mechanisms.

Ceramide plays an important role in the transition of cells into replicative senescence [26,28,46], a state in which mitotic cells irreversibly stop dividing and start secreting pro-inflammatory cytokines and tissue-modifying factors [39,40]. Our *in vitro* experiments indicate that the increased ceramide biosynthesis evoked by D-meth accelerates progression of a senescent phenotype characterized by lowered proliferation, altered cell morphology and heightened expression of β-Gal and other senescence-associated markers. Among such markers is a group of genes encoding for protein regulators of cell cycle (p53, p16, p21 and IGF-1) and inflammation (IL-6 and TNF-α), which we found to be abnormally high in skeletal muscle and other peripheral tissues of rats that voluntarily take D-meth. Importantly, this same panel of genes is known to be elevated in tissues of elderly persons [47,48,49] and is thought to participate in chronic inflammatory conditions that are typical of old age [40]. The premature occurrence of such conditions is a hallmark of D-meth abuse in humans, which cannot be explained by the psychostimulant and sympathomimetic properties of this drug [17,18], but is consistent with its ability to elevate ceramide levels. Indeed, overactive ceramide signaling has been implicated in several age-related pathologies—including coronary artery atherosclerosis, liver steatosis and pulmonary fibrosis [50,51,52]—which are frequently documented in autopsy reviews of deceased D-meth users [16,17,18]. Collectively, the available data suggest that the rapid health decline caused by D-meth is due to a ceramide-mediated acceleration of genetic programs that are also engaged during chronic inflammation and aging. Pharmacological strategies aimed at normalizing ceramide signaling—for example by modulating NF-κB recruitment (which include drugs that are already available in the clinic, such as 5-aminosalicylic acid and thalidomide) or SPT activity—might slow down this pathogenic process and facilitate detoxification of D-meth users.

## Supporting Information

S1 Fig D-meth intake in rats self-administering the drug. (A) Daily D-meth intake and (B) number of active hole responses vs non-active hole responses. After 8 days of self-administration, the rats were sacrificed and tissues were collected to perform the lipidomic analyses reported in Fig. 1.
(TIF)

S2 Fig Effects of acute D-meth administration (mg-kg^-1^, i.p.) on (A) ceramide levels, (B) ceramide synthase 6 (CerS) mRNA levels and (C) ceramide synthase activity in rat skeletal muscle. *P<0.05, **P<0.01, ***P<0.001, ANOVA followed by Bonferroni post hoc test.
(TIF)

S3 Fig D-meth stimulation of ceramide production in immortalized cultures of MEF. Time-course of the effects of vehicle (○) or D-meth (1mM, ■) on **(A)** dihydro-sphingosine, **(B)** dihydro-ceramides, **(C)** ceramides and **(D)** sphingomyelins. The panel on the left-hand side shows key intermediates in *de novo* ceramide biosynthesis, which were targeted in the present analyses. Values are expressed as mean±s.e.m., n = 3 for each experimental time point. *P<0.05, **P<0.01, ***P<0.001, two-way ANOVA followed by Bonferroni *post hoc* test.
(TIF)

S4 Fig D-meth stimulation of ceramide production in serum-differentiated C2C12 myotubes. Concentration dependence of the effects of D-meth on **(A)** dihydro-sphingosine, **(B)** dihydro-ceramide (d18:0/16:0), **(C)** ceramide (d18:1/16:0), and **(D)** sphingomyelin (d18:0/16:0). C2C12 cells were differentiated with 2% horse serum for 8 days and then treated with D-meth (0.1–1 mM) for 24 h. Values are expressed as mean±s.e.m., n = 3 for each experimental point. **P<0.01, ***P<0.001, one-way ANOVA followed by Bonferroni *post hoc* test.
(TIF)

S5 Fig Effects of D-Meth (0, 0.5, 1 mM; 48 h) or cisplatin (20 and 50 μM; 48 h) on primary MEF viability. Cell viability was assessed using the MTT assay. Values are expressed as mean±s.e.m. n = 4 for each experimental point. ***P<0.001, one-way ANOVA followed by Bonferroni *post hoc* test.
(TIF)

S6 Fig The NF-κB inhibitor thalidomide reverts the effect of D-meth on the transcription of enzymes of ceramide biosynthesis in immortalized MEF cultures. Effects of vehicle (dimethylsulfoxide, 0.1%), D-meth (1 mM), thalidomide (25 μM) or a combination of D-meth plus thalidomide on gene transcription. Abbreviations: SPT, serine palmitoyl-coenzyme A transferase; CerS, ceramide synthase. Cells were exposed to the drugs for 24 h. Values are expressed as mean±s.e.m. (n = 3). *P<0.05, **P<0.01, ***P<0.001, versus vehicle; °°*P<0*.*01*, °°°*P<0*.*001* versus D-Meth. One-way ANOVA followed by *post hoc* test.
(TIF)

S7 Fig Effect of siRNA-mediated gene silencing on the transcription of NF-κB p65 subunit in cultures of immortalized MEF. NF-κB p65 mRNA was quantified by qRT-PCR. Values are expressed as mean±s.e.m. (n = 3). ***P<0.001, versus vehicle. One-way ANOVA followed by Bonferroni *post hoc* test.
(TIF)

S8 Fig NF-κB silencing inhibits D-meth-induced activation of ceramide biosynthesis in cultures of immortalized MEF. Effects of vehicle or D-meth (1 mM, 24 h) with or without p65 siRNA on **(A)** dihydro-ceramides, **(B)** ceramides and **(C)** sphingomyelins. Values are expressed as mean±s.e.m. (n = 3). **P<0.01, ***P<0.001, versus vehicle;°°*P<0*.*01*, °°°*P<0*.*001* versus D-Meth. One-way ANOVA followed by Bonferroni *post hoc* test.
(TIF)

S9 Fig NF-κB silencing suppresses D-meth-induced induction of enzymes of *de novo* ceramide biosynthesis in cultures of immortalized MEF. Effects of vehicle or D-meth (1 mM) with or without p65 siRNA on gene transcription. SPT, serine palmitoyl-coenzyme A transferase; CerS, ceramide synthase. Values are expressed as mean±s.e.m. (n = 3). *P<0.05, **P<0.01, ***P<0.001, versus vehicle;°°*P<0.01*, °°°*P<0.001* versus D-Meth. One-way ANOVA followed by Bonferroni *post hoc* test.
(TIF)

S10 Fig Pharmacological inhibition or genetic silencing of NF-κB prevents D-meth-induced induction of senescence- and inflammation-associated markers in cultures of immortalized MEF. mRNA levels in MEF treated with **(A)** thalidomide (25 μM) or **(B)** p65 siRNA in combination with D-meth (1mM, 24 h). Abbreviations: IL-6, interleukin-6; TNF-α, tumor necrosis factor-α; *P<0.05, **P<0.01, ***P<0.001, versus vehicle;°°*P<0*.*01*, °°°*P<0*.*001* versus D-Meth. One-way ANOVA followed by Bonferroni *post hoc* test.
(TIF)

S1 Materials and Methods (DOCX)

S1 Table Levels of various lipid species in brain regions of rats self-administering D-meth and yoked control rats. Abbreviations: DAG, diacylglycerol; DHC, dihydro-ceramide; DH-Sphm, dihydro-sphingomyelin; MAG, monoacylglycerol; M-FA, monounsaturated fatty acid; PC, phosphatidylcholine; PE, phosphatidylethanolamine; P-FA, polyunsaturated fatty acid; PG, phosphatidylglycerol; PI, phosphatidylinositol; PS, phosphatidylserine; S-FA, saturated fatty acid; Sphm, sphingomyelin; TAG, triacylglycerol. Values are expressed as mean±s.e.m. *P<0.05, P; ***P<0.001; N.D., not detected; planned comparisons obtained from Proc Mixed analysis with False Discovery Rate correction for multiple comparisons (n = 12 in D-meth group and 6 in control group).
(DOCX)

S2 Table Levels of ceramide species in brain regions of rats self-administering D-meth and yoked control rats. Values are expressed as mean±s.e.m. *P<0.05, P; N.D., not detected; planned comparisons obtained from Proc Mixed analysis with False Discovery Rate correction for multiple comparisons (n = 6 in D-meth group and 6 in control group).
(DOCX)

S3 Table Levels of various lipid species in organs and tissues of rats self-administering D-meth and yoked control rats. Abbreviations: DAG, diacylglycerol; DHC, dihydroceramide; DH-Sphm, dihydro-sphingomyelin; MAG, monoacylglycerol; M-FA, monounsaturated fatty acid; PC, phosphatidylcholine; PE, phosphatidylethanolamine; P-FA, polyunsaturated fatty acid; PG, phosphatidylglycerol; PI, phosphatidylinositol; PS, phosphatidylserine; S-FA, saturated fatty acid; Sphm, sphingomyelin; TAG, triacylglycerol. Values are expressed as mean±s.e.m. *P<0.05; N.D., not detected; planned comparisons obtained from Proc Mixed analysis with False Discovery Rate correction for multiple comparisons (n = 6 in D-meth group and 6 in control group).
(DOCX)

S4 Table Levels of ceramide species in peripheral tissues of rats self-administering D-meth and yoked control rats. Values are expressed as mean±s.e.m. *P<0.05, **P<0.01; ***P<0.001; N.D., not detected; planned comparisons obtained from Proc Mixed analysis with False Discovery Rate correction for multiple comparisons; (n = 6 in D-meth group and 6 in control group).
(DOCX)

S5 Table Levels of mRNAs encoding for enzymes of *de novo* ceramide biosynthesis in brain regions of rats self-administering D-meth and yoked control rats. Values are expressed as mean±s.e.m. of mRNA/GAPDH*1000. *P<0.05, P; **P<0.01; ***P<0.001; N.D., not detected; two-tailed Student’s t test (n = 6–12).
(DOCX)

S6 Table Levels of mRNAs encoding for enzymes of *de novo* ceramide biosynthesis in peripheral tissues of rats self-administering D-meth and yoked control rats. Values are expressed as mean±s.e.m. of mRNA/GAPDH*1000. *P<0.05, P; **P<0.01; ***P<0.001; N.D., not detected; two-tailed Student’s t test (n = 6–12).
(DOCX)

S7 Table Levels of mRNAs encoding for Tumor Necrosis Factor-α (TNF-α) and interleukin-6 in peripheral tissues of rats self-administering D-meth and yoked control rats. Values are expressed as mean±s.e.m. of mRNA/GAPDH*1000. *P<0.05, P; **P<0.01; ***P<0.001; two-tailed Student’s *t* test (n = 6–12).
(DOCX)

S8 Table Sequences of RT-PCR primers utilized in the present study. (DOCX)
