# Supplementary figures and images for: Methamphetamine Accelerates Cellular Senescence through Stimulation of De Novo Ceramide Biosynthesis
Source: PLoS One. 2015 Feb 11;10(2):e0116961. doi: 10.1371/journal.pone.0116961 (PMC4324822; doi:10.1371/journal.pone.0116961)

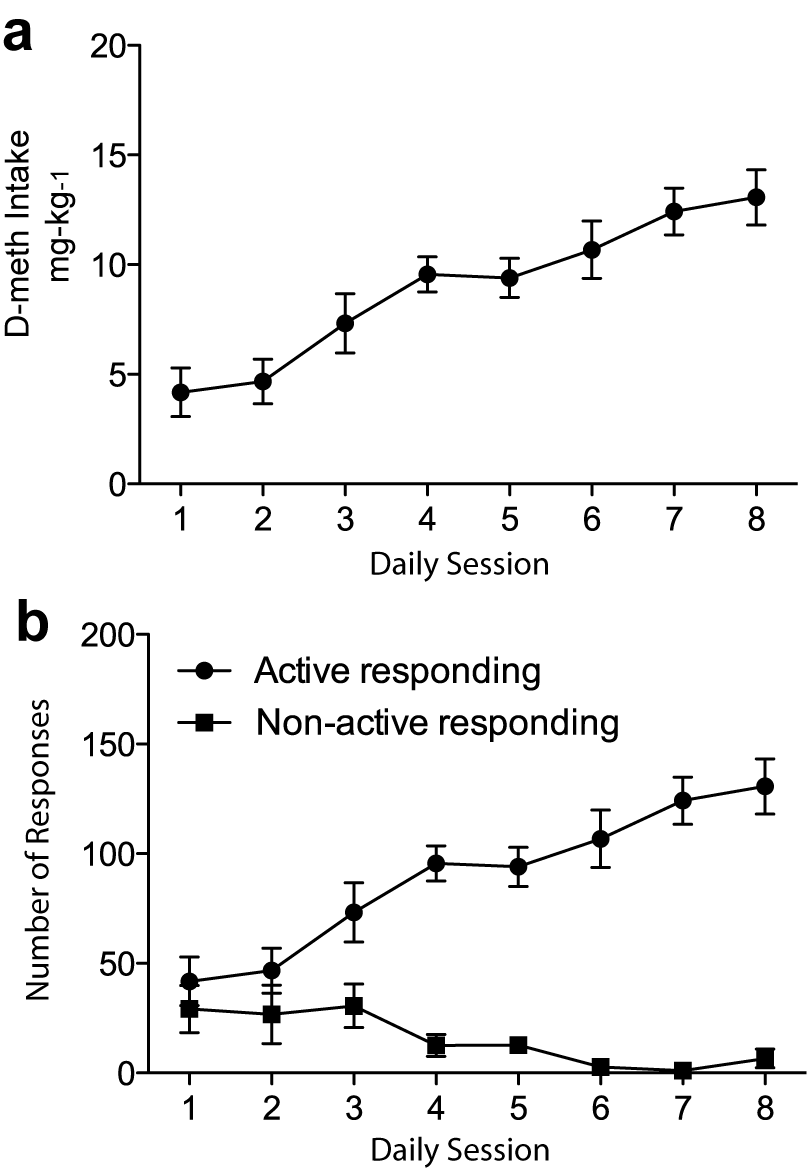

Supplement: S1 Fig — (A) Daily D-meth intake and (B) number of active hole responses vs non-active hole responses. After 8 days of self-administration, the rats were sacrificed and tissues were collected to perform the lipidomic analyses reported in Fig. 1. (TIF) [file pone.0116961.s001.tif]

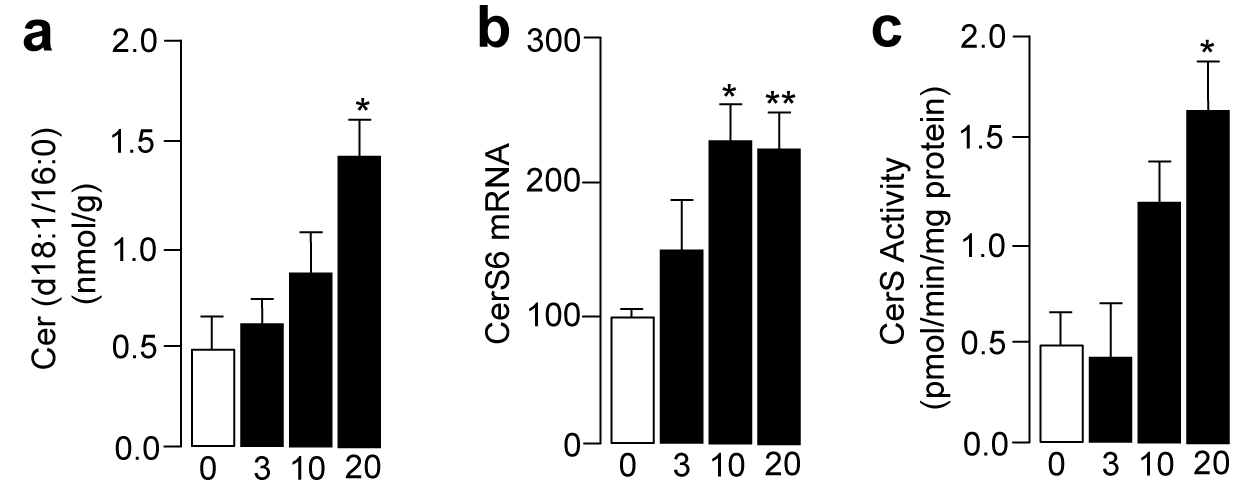

Supplement: S2 Fig — *P<0.05, **P<0.01, ***P<0.001, ANOVA followed by Bonferroni post hoc test. (TIF) [file pone.0116961.s002.tif]

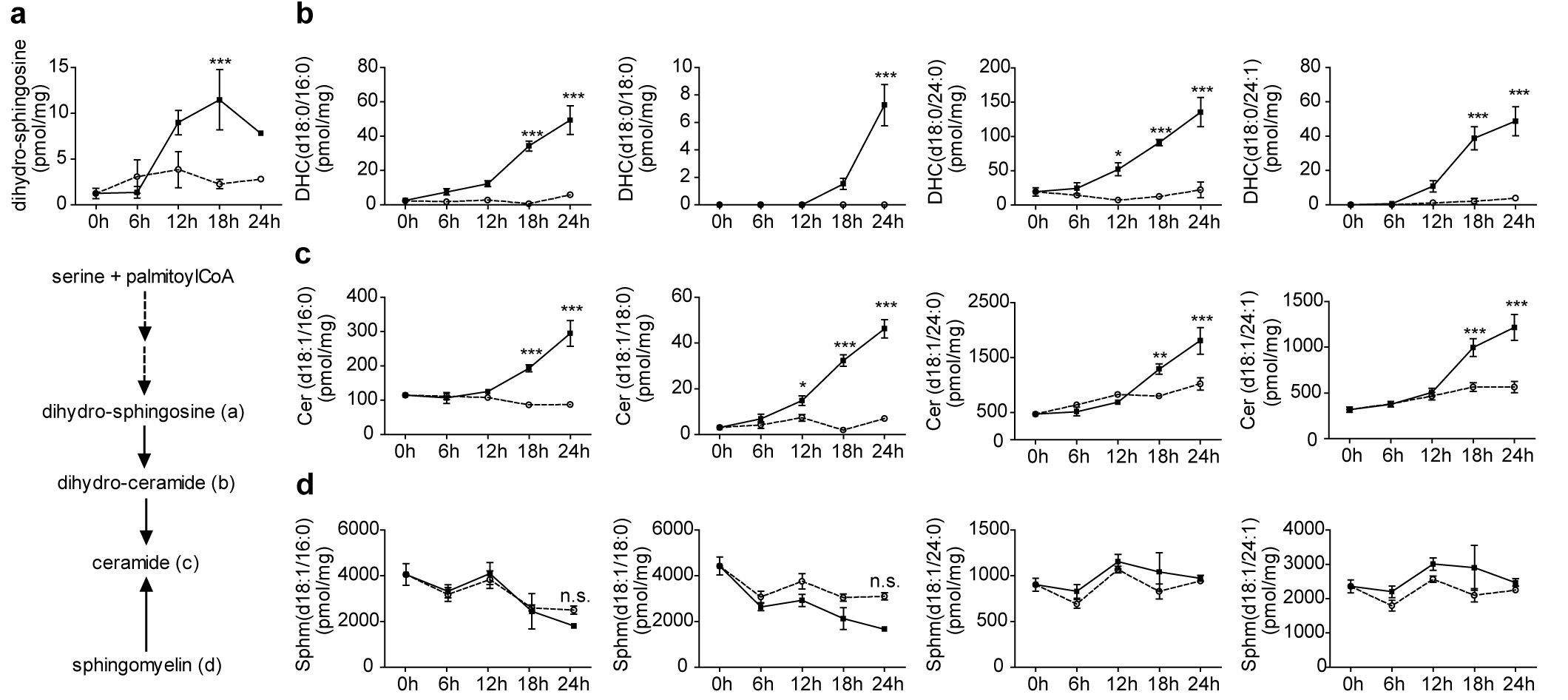

Supplement: S3 Fig — Time-course of the effects of vehicle (○) or D-meth (1mM, ■) on (A) dihydro-sphingosine, (B) dihydro-ceramides, (C) ceramides and (D) sphingomyelins. The panel on the left-hand side shows key intermediates in de novo ceramide biosynthesis, which were targeted in the present analyses. Values are expressed as mean±s.e.m., n = 3 for each experimental time point. *P<0.05, **P<0.01, ***P<0.001, two-way ANOVA followed by Bonferroni post hoc test. (TIF) [file pone.0116961.s003.tif]

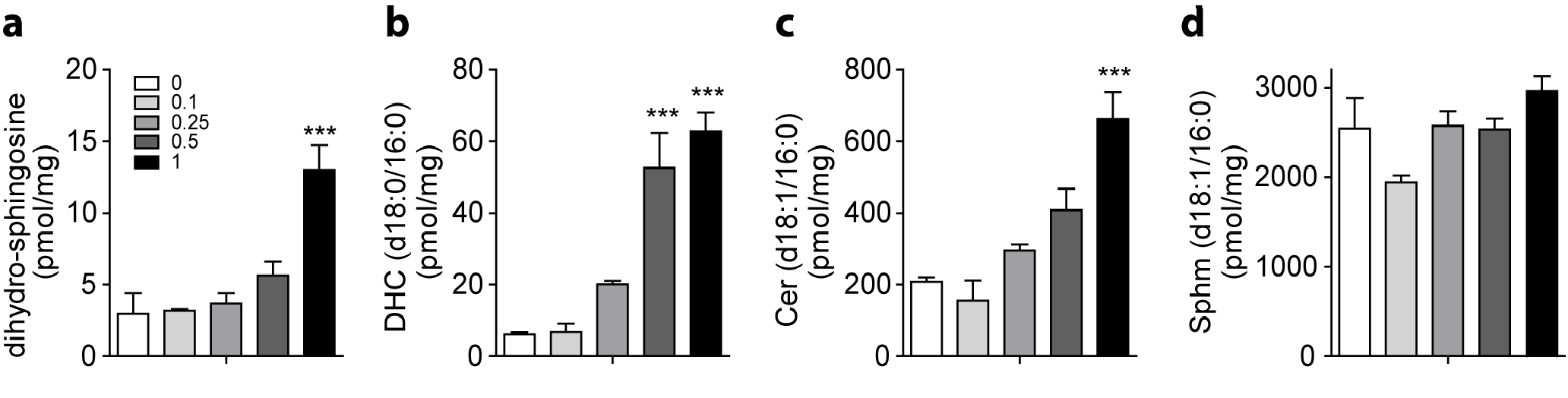

Supplement: S4 Fig — Concentration dependence of the effects of D-meth on (A) dihydro-sphingosine, (B) dihydro-ceramide (d18:0/16:0), (C) ceramide (d18:1/16:0), and (D) sphingomyelin (d18:0/16:0). C2C12 cells were differentiated with 2% horse serum for 8 days and then treated with D-meth (0.1–1 mM) for 24 h. Values are expressed as mean±s.e.m., n = 3 for each experimental point. **P<0.01, ***P<0.001, one-way ANOVA followed by Bonferroni post hoc test. (TIF) [file pone.0116961.s004.tif]

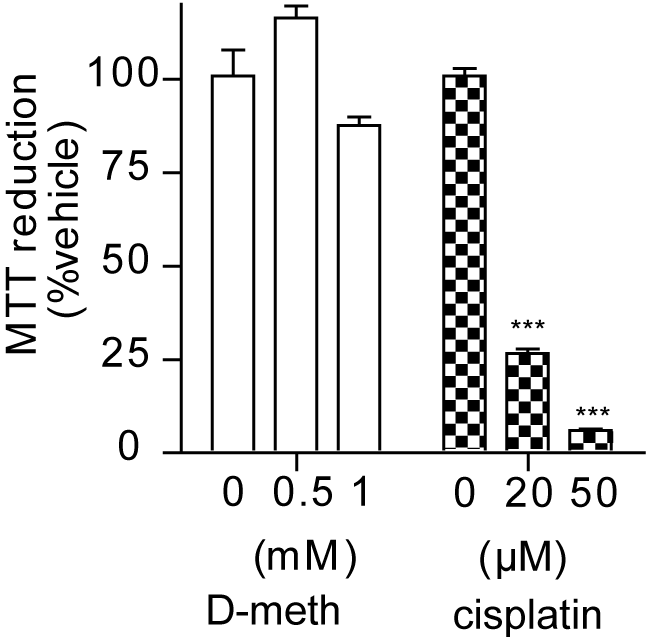

Supplement: S5 Fig — Cell viability was assessed using the MTT assay. Values are expressed as mean±s.e.m. n = 4 for each experimental point. ***P<0.001, one-way ANOVA followed by Bonferroni post hoc test. (TIF) [file pone.0116961.s005.tif]

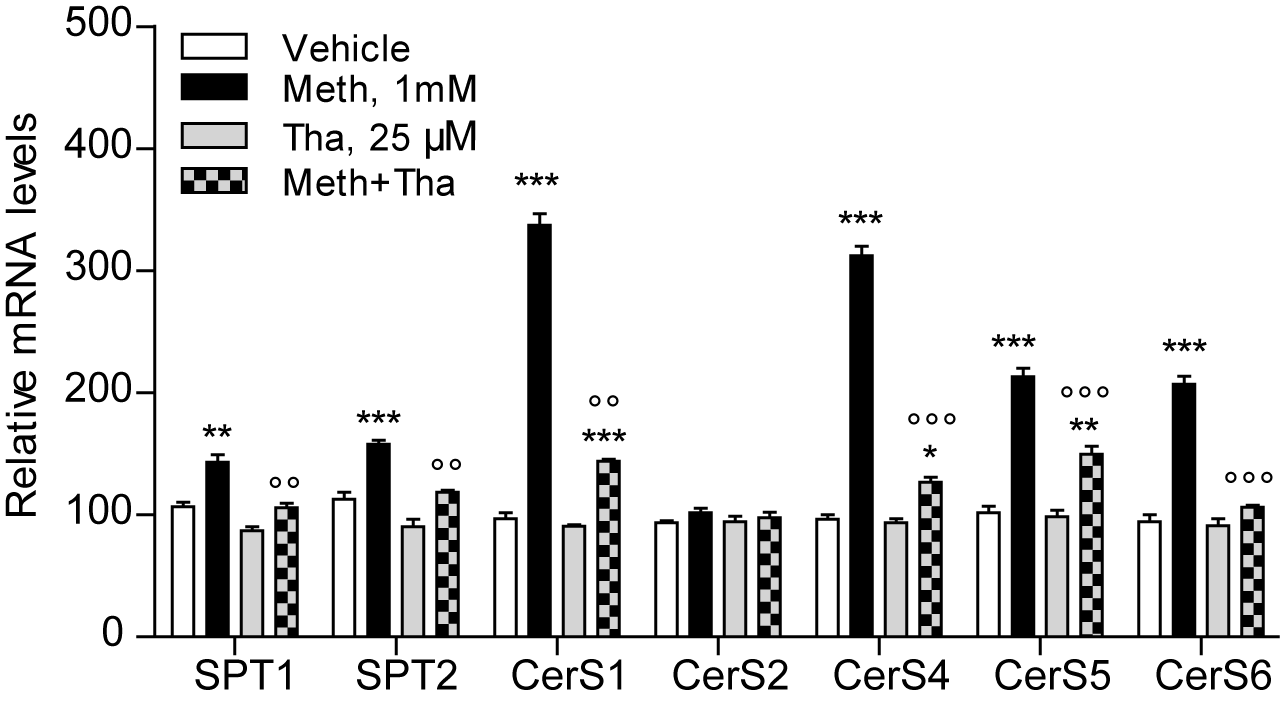

Supplement: S6 Fig — Effects of vehicle (dimethylsulfoxide, 0.1%), D-meth (1 mM), thalidomide (25 μM) or a combination of D-meth plus thalidomide on gene transcription. Abbreviations: SPT, serine palmitoyl-coenzyme A transferase; CerS, ceramide synthase. Cells were exposed to the drugs for 24 h. Values are expressed as mean±s.e.m. (n = 3). *P<0.05, **P<0.01, ***P<0.001, versus vehicle; °°P<0.01, °°°P<0.001 versus D-Meth. One-way ANOVA followed by post hoc test. (TIF) [file pone.0116961.s006.tif]

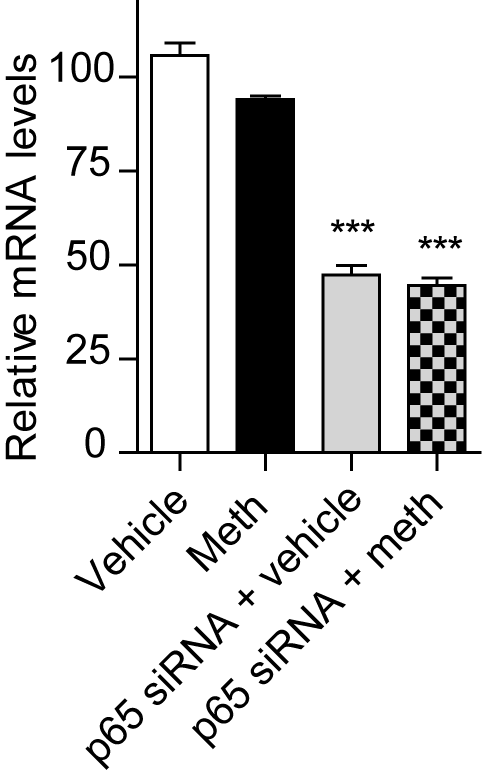

Supplement: S7 Fig — NF-κB p65 mRNA was quantified by qRT-PCR. Values are expressed as mean±s.e.m. (n = 3). ***P<0.001, versus vehicle. One-way ANOVA followed by Bonferroni post hoc test. (TIF) [file pone.0116961.s007.tif]

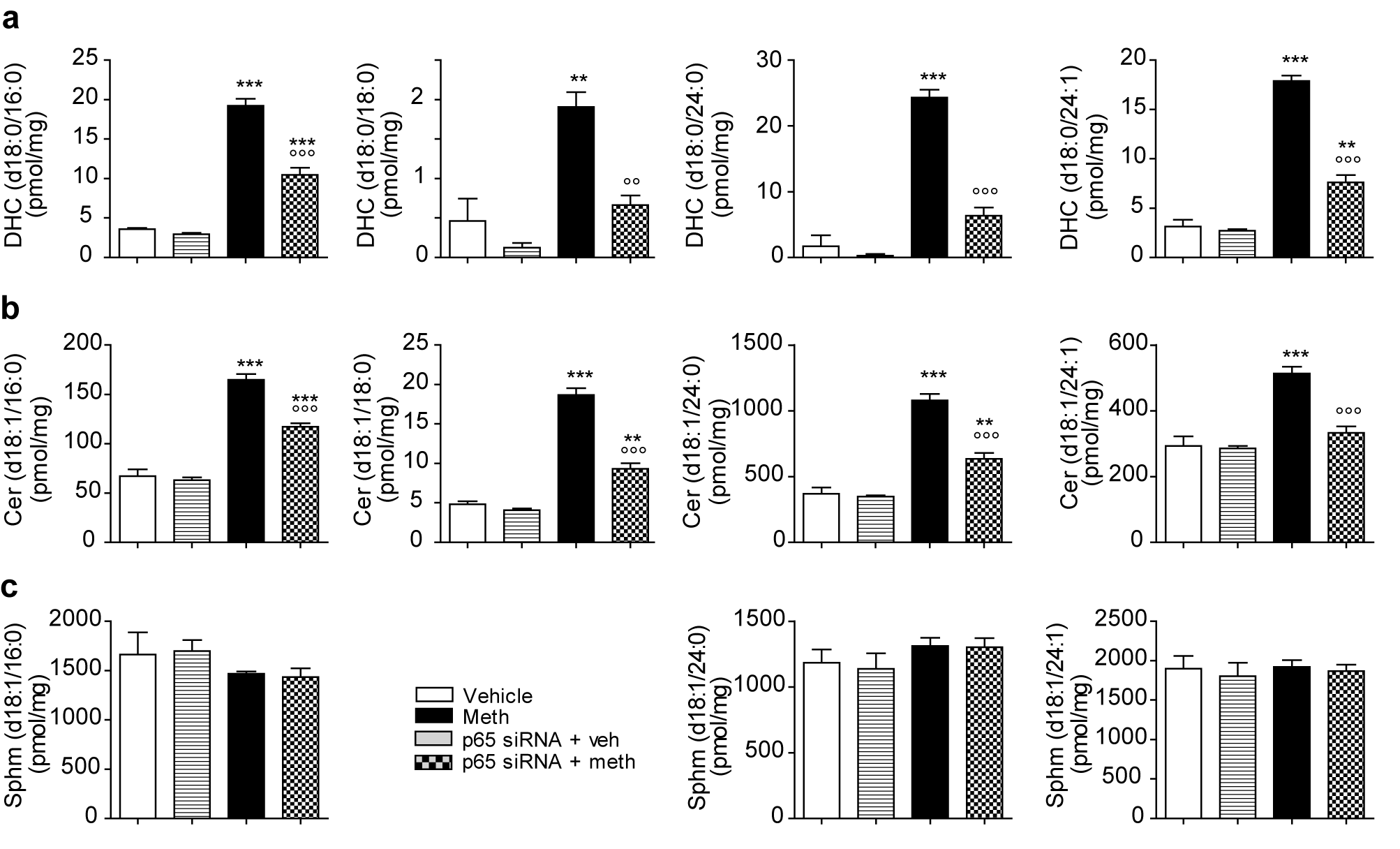

Supplement: S8 Fig — Effects of vehicle or D-meth (1 mM, 24 h) with or without p65 siRNA on (A) dihydro-ceramides, (B) ceramides and (C) sphingomyelins. Values are expressed as mean±s.e.m. (n = 3). **P<0.01, ***P<0.001, versus vehicle;°°P<0.01, °°°P<0.001 versus D-Meth. One-way ANOVA followed by Bonferroni post hoc test. (TIF) [file pone.0116961.s008.tif]

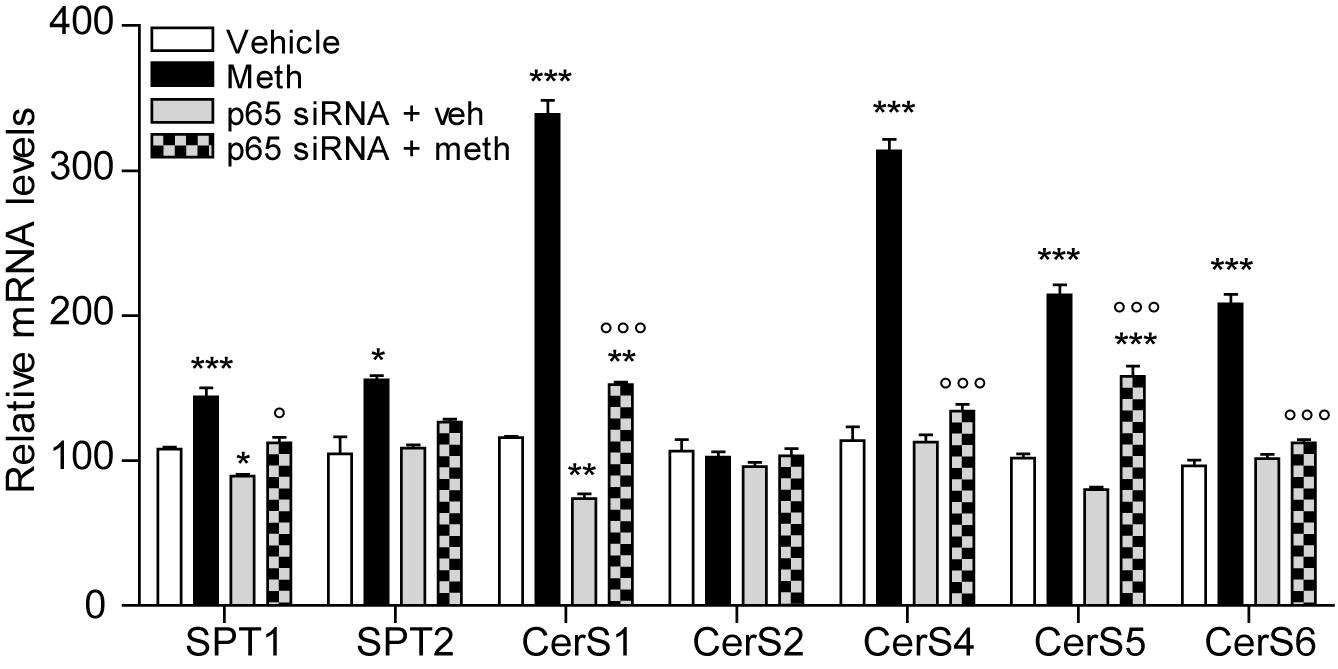

Supplement: S9 Fig — Effects of vehicle or D-meth (1 mM) with or without p65 siRNA on gene transcription. SPT, serine palmitoyl-coenzyme A transferase; CerS, ceramide synthase. Values are expressed as mean±s.e.m. (n = 3). *P<0.05, **P<0.01, ***P<0.001, versus vehicle;°°P<0.01, °°°P<0.001 versus D-Meth. One-way ANOVA followed by Bonferroni post hoc test. (TIF) [file pone.0116961.s009.tif]

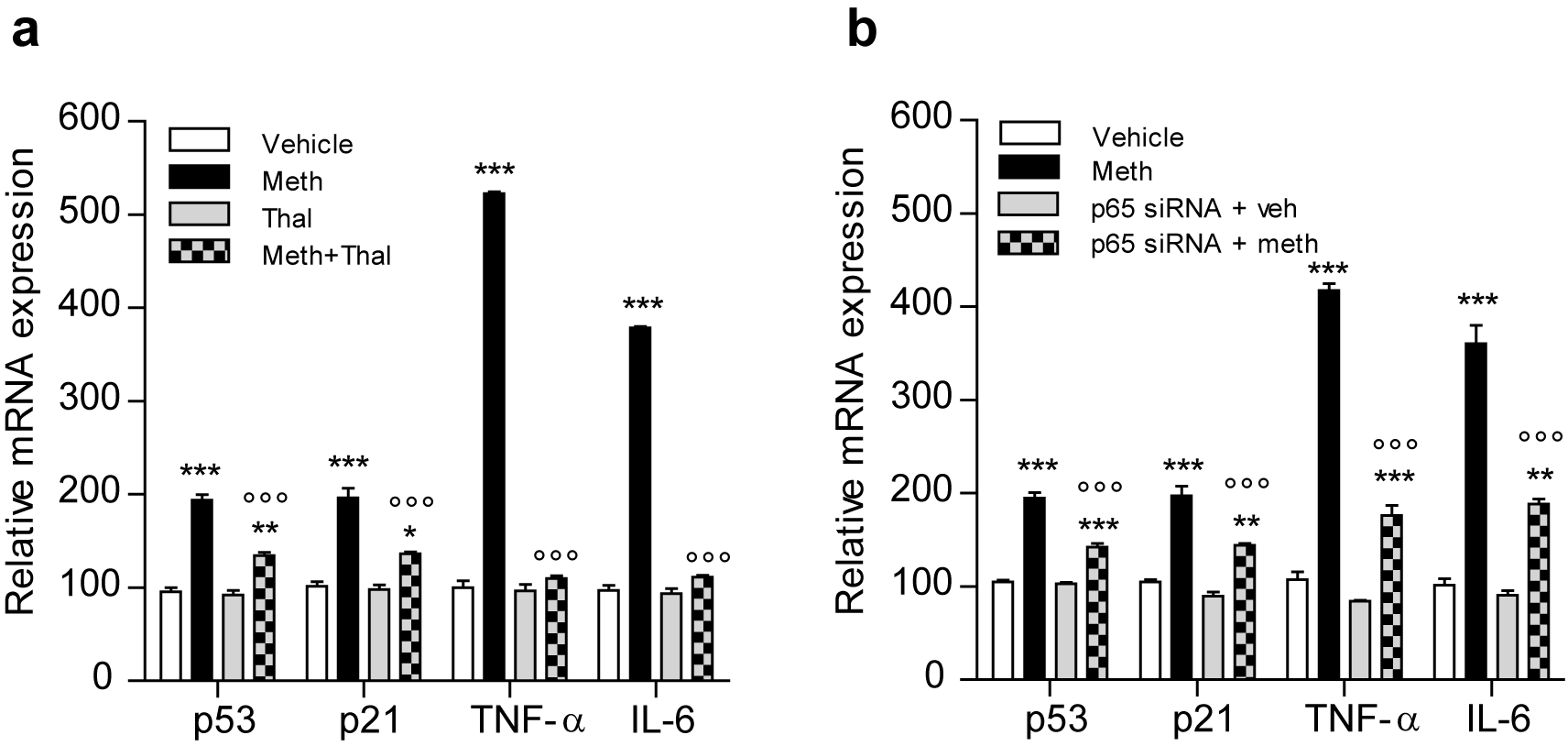

Supplement: S10 Fig — mRNA levels in MEF treated with (A) thalidomide (25 μM) or (B) p65 siRNA in combination with D-meth (1mM, 24 h). Abbreviations: IL-6, interleukin-6; TNF-α, tumor necrosis factor-α; *P<0.05, **P<0.01, ***P<0.001, versus vehicle;°°P<0.01, °°°P<0.001 versus D-Meth. One-way ANOVA followed by Bonferroni post hoc test. (TIF) [file pone.0116961.s010.tif]
