# Supplementary material for: Methamphetamine Accelerates Cellular Senescence through Stimulation of De Novo Ceramide Biosynthesis
Source: PLoS One. 2015 Feb 11;10(2):e0116961. doi: 10.1371/journal.pone.0116961 (PMC4324822; doi:10.1371/journal.pone.0116961)
Supplement: S1 Materials and Methods — (DOCX) [file pone.0116961.s011.docx]

**Supporting Information**

**Materials and methods**

**Cell cultures**

Immortalized MEF were purchased from American Type Culture Collection (Manassas, VA) and cultured in Dulbecco’s Modified Eagle’s Medium (DMEM) containing 10% fetal bovine serum, 2mM L-glutamine, 100 U/ml penicillin and streptomycin at 37°C and 5% CO_2_. Murine C2C12 cells were a kind gift of Dr. Maria Pennuto’s group (Italian Institute of Technology, Genova, Italy) and were grown in Dulbecco’s Modified Eagle’s Medium (DMEM) containing 10% fetal bovine serum, 2mM L-glutamine, 100 U/ml penicillin and streptomycin. Cells were differentiated into myotubes as follows: they were seeded at 5x10^6^ cells/well in a 6-wells format and regular media was substituted by differentiation medium to induce differentiation (DMEM containing 2% horse serum, 100 U/ml penicillin and streptomycin, referred as differentiation medium or DM). To obtain myotubes, cells were cultured for 8 days in DM before treatment with D-meth. Primary MEF cultures were prepared from C57BL/6 mouse embryos, as described [48]. Briefly, pregnant mice at day 13 post coitum were killed and the uteri were removed. Each embryo was separated from the placenta, and head and visceral tissues were dissected out. The remaining body was minced in phosphate-buffered saline (PBS) and incubated with 0.1 mM trypsin/1 mM EDTA at 37°C for 15 min. Two volumes of Dulbecco’s Modified Eagle Medium (DMEM) containing 10% fetal bovine serum (FBS) were added and let stand for 5 min to let set down large pieces of unbroken tissues. The supernatant was removed, centrifuged at 200xg for 5 min and suspended in fresh DMEM containing 10% FBS. Cells were cultured at 37°C with 5% CO_2_.

**Lipid extractions**

Lipid extractions were carried out as described [49]. Briefly, frozen tissue samples were weighed and homogenized in cold methanol containing internal standards. Lipids were extracted by adding chloroform and water (2/1, vol/vol) and fractionated through open-bed silica gel columns by progressive elution with chloroform/methanol mixtures. Fractions eluted from the columns were dried under nitrogen, reconstituted in chloroform/methanol (1:4, vol/vol; 0.1 ml) and subjected to LC/MS analyses. Lipid extractions from cells in cultures were conducted as follows. Cells were washed with ice-cold PBS and scraped into 0.5 ml of methanol/water (1/1, vol/vol) containing internal standards. Protein concentration was measured using the bicinchoninic acid (BCA) assay (Pierce, Rockford, IL, USA). Lipids were extracted with chloroform/methanol (2/1, vol/vol; 1 ml). The organic phases were collected, dried under nitrogen and dissolved in methanol for LC/MS analyses.

**Lipid analyses**

*Fatty acids.* Fatty acids were identified and quantified using an Agilent 1100 liquid chromatograph coupled to a 1946D mass detector equipped with an ESI interface (Agilent Technologies, Palo Alto, CA). A reversed-phase XDB Eclipse C18 column (50x4.6 mm i.d., 1.8 μm, Zorbax, Agilent Technologies) was eluted with a linear gradient from 90% to 100% of A in B for 2.5 min at a flow rate of 1.5 ml/min with column temperature at 40˚C. Mobile phase A consisted of methanol containing 0.25% acetic acid and 5 mM ammonium acetate; mobile phase B consisted of water containing 0.25% acetic acid and 5 mM ammonium acetate. Mass detection was in the negative ionization mode, capillary voltage was set at -4.0 kV and fragmentor voltage was 120 V. Nitrogen was used as drying gas at a flow rate of 13 l-min^-1^ and a temperature of 350˚C. Nebulizer pressure was set at 60 pounds per square inch. For quantification purposes, the deprotonated pseudo-molecular ions [M-H]- of the fatty acids were monitored in the selected ion-monitoring mode (SIM), using d^8^-arachidonic acid (Cayman Chemical, Ann Arbor, MI) as internal standard (m/z = 311.3). Commercially available fatty acids (Nu-Chek Prep, Elysian, MN, Cayman Chemical or Sigma-Aldrich, St Louis, MO) were used as references.

*Monoacylglycerols (MGs).* We used an Agilent 1100-LC system (Agilent Technologies, Palo Alto, CA) coupled to a 1946D-MS detector equipped with an ESI interface (Agilent Technologies). MGs were separated on a XDB Eclipse C18 column (50 × 4.6 mm i.d., 1.8 μm; Zorbax; Agilent Technologies). They were eluted with a gradient of methanol in water (from 85% to 90% methanol in 2.0 min and 90% to 100% in 3.0 min) at a flow rate of 1.5 ml-min^-1^. Column temperature was kept at 40°C. MS detection was in the positive ionization mode, capillary voltage was set at 3 kV, and fragmentor voltage was 120 V. Nitrogen was used as drying gas at a flow rate of 13 l-min^-1^ and a temperature of 350°C. Nebulizer pressure was set at 60 psi. Commercial MGs were used as reference standards. For quantification purposes, we monitored the Na+ adducts of the molecular ions [M+Na]+ in SIM mode, using (1,3)heptadecanoyl-*sn*- glycerol (m/z = 367) as an internal standard.

*Diacylglycerols (DGs).* We used an Agilent 1100-LC system coupled to a MS detector Ion-Trap XCT interfaced with ESI (Agilent Technologies). DG species were separated using a XDB Eclipse C18 column (50 x 4.6 mm i.d., 1.8 μm, Zorbax), eluted by a gradient of methanol in water (from 85% to 90% methanol in 2.5 min) at a flow rate of 1.5 ml-min^-1^. Column temperature was kept at 40˚C. The capillary voltage was set at 4.0 kV and skimmer voltage at 40 V. Nitrogen was used as drying gas at a flow rate of 12 l-min^-1^, temperature at 350°C, and nebulizer pressure at 80 psi. Helium was used as collision gas, and fragmentation amplitude was set at 1.2 V. DGs were identified in the positive ionization mode based on their retention times and MS3 properties, using synthetic standards as references. Multiple reaction monitoring was used to acquire full-scan tandem MS spectra of selected DG ions. Extracted ion chromatograms were used to quantify isobaric DG species and dinonadecadienoin (m/z 667.8 > 367.5), which was used as an internal standard.

*Triacylglycerols (TGs).* We used an Agilent 1100-LC system coupled to a MS detector Ion-Trap XCT interfaced with atmospheric pressure chemical ionization (Agilent Technologies). Lipids were separated on a Poroshell 300SB C18 column (2.1x75 mm i.d., 5μm, Agilent Technologies) at 50°C. A linear gradient of methanol in water containing 5 mM ammonium acetate and 0.25% acetic acid (from 85% to 100% of methanol in 4 min) was applied at a flow rate of 1 ml-min^-1^. MS detection was set in positive mode. Corona discharge needle voltage set at 4 kV. Capillary voltage was 4.0 kV, skim1 40 V, and capillary exit at 118 V. Nitrogen was used as drying gas at a flow rate of 10 l-min^-1^, temperature of 350°C, nebulizer pressure of 50 PSI and vaporization temperature at 400°C. Helium was used as collision gas. TGs were quantified by integrating the area of the total ion current (m/z 700-900) at a selected interval of retention time (from 4 to 5 min), using trinonadecenoin (m/z 944.8, Nu-Chek Prep) as an internal standard.

*Glycerophospholipids.* Phospholipids molecular species were analyzed by tandem mass spectrometry, using an Agilent 1100 LC system coupled to an ESI-ion-trap XCT mass detector. A reversed-phase Poroshell 300SB C18 column (2.1x75 mm i.d., 5 μm, Agilent) was eluted with a linear gradient from 85% to 100% of mobile phase A in B in 5 min at a flow rate of 1.0 ml-min^-1^ with column temperature at 50˚C. Mobile phase composition was as described above. The capillary voltage was set at 4.0 kV and skimmer voltage at -40 V. Nitrogen was utilized as drying gas at a flow rate of 10 l-min^-1^, temperature at 350˚C and nebulizer pressure at 60 psi. Helium was the collision gas and fragmentation amplitude was set at 1.2 V. Mass detection was in the negative ionization mode and was controlled by the Agilent/Bruker Daltonics software version 5.2. Synthetic 1,2-diheptadecanoyl-*sn*-glycero-3-phosphoethanolamine, 1,2-diheptadecanoyl-*sn*-glycero-3-phosphocholine, 1,2-diheptadecanoyl-*sn*-glycero-3-phosphoglycerol, 1,2-diheptadecanoyl-sn-glycero-3-phosphoserine,1,2-diheptadecanoyl-*sn*-glycero-3-phosphoinositol (Avanti Polar Lipids, Alabaster, AL) were used as internal standards.

*Sphingolipids.* Sphingolipid analyses were carried out using either of the following two methods. In one set of experiments, we used an Agilent LC coupled to an ESI-ion-trap XCT mass spectrometer. Dihydroceramides and ceramides were separated on a Poroshell 300 SB C18 column (2.1 x 75 mm i.d., 5 μm; Agilent Technologies) maintained at 30°C. A linear gradient of methanol in water containing 5 mM ammonium acetate and 0.25% acetic acid (from 80% to 100% of methanol in 3 min) was applied at a flow rate of 1 ml/min. Detection was in the positive mode using the following fragmentation transitions: d18:1/16:0 [M-H_2_O+H]^+^ (*m/z* = 520.5>264.3), d18:0/16:0 [M+H]^+^ (*m/z* = 540.5>522.5), d18:1/18:0 [M-H_2_O+H]^+^ (*m/z* = 548.5>264.3), d18:0/18:0 [M+H]^+^ (*m/z* = 568.5>550.5), d18:1/24:0 [M-H_2_O+H]^+^ (*m/z* = 632.8>264.3), d18:0/24:0 [M+H]^+^ (*m/z* = 652.6>634.8), d18:1/24:1 [M-H_2_O+H]^+^ (*m/z* = 630.8>264.3), d18:0/24:1 [M+H]^+^ (*m/z* = 650.6>632.8) and d18:1/12:0 [M-H_2_O+H]+ (*m/z* = 464.5>264.3) (Avanti Polar Lipids, Alabaster, AL) (Avanti Polar Lipids, Alabaster, AL), which was included as an internal standard. Sphingomyelins were separated as detailed for glycerophospholipids (see above) and were analyzed in positive ionization mode using the following multiple-ion reactions: d18:1/16:0 [M+H]^+^ (*m/z* = 703.8>644.8), d18:1/18:0 [M+H]^+^ (*m/z* = 731.8>672.8), d18:1/24:0 [M+H]^+^ (*m/z* = 815.8>756.8), d18:1/24:1 [M+H]^+^ (*m/z* = 813.8> 754.8) and d18:1/12:0 [M+H]+ (*m/z* = 647.8>588.8), which was included as an internal standard (Avanti Polar Lipids). In a second set of experiments, ceramides, sphingosine, sphinganine and sphingomyelins were analyzed by LC-MS/MS using a Waters Acquity UPLC coupled with a Waters Xevo TQMS and interfaced with ESI. Separation was done on a Waters Acquity BEH C18 1.7µm column (2.1 x 50 mm) at 60 °C. A step gradient of 0.1% formic acid in acetonitrile/water (20:80 v/v) as solvent A and 0.1 formic acid in acetonitrile/isopropyl alcohol (20:80 v/v) as solvent B was applied at a flow rate of 0.4 mL/min. Capillary voltage was 3.5 kV and cone voltage was 25 V. The source temperature and desolvation temperatures were set at 120°C and 600 °C respectively. Desolvation gas and cone gas (nitrogen) flow were 800 and 20 l/h, respectively. Detection was in the positive mode using the following fragmentation transitions: d18:1/14:0 (*m/z* 510.5>492.5>264.3), d18:1/16:0 (*m/z* = 538.5> 520.3>264.3), d18:1/18:0 (*m/z* 566.5>548.3>264.3), d18:1/24:0 (*m/z* = 650.5>632.3>264.3), d18:1/24:1 (*m/z* = 648.5>630.3>264.3), using d18:1/14:0 as internal standard (*m/z* = 482.5>464.5>264.3).

**D-Methamphetamine measurements**

Methamphetamine was measured using an Agilent 1100 liquid chromatograph coupled to a 1946D mass detector equipped with an ESI interface (Agilent). A reversed-phase XDB Eclipse C18 column (50x4.6 mm i.d., 1.8 μm, Zorbax, Agilent) was eluted with an escalating gradient from 0% to 100% of A to B for 4.5 min at a flow rate of 1.0 ml/min with column temperature at 22˚C. Mobile phase A consisted of water containing 0.25% acetic acid and 5 mM ammonium acetate; mobile phase B consisted of methanol containing 0.25% acetic acid and 5 mM ammonium acetate. MS detection was in the positive ionization mode, capillary voltage was set at 3 kV, and fragmentor voltage was 120 V. Nitrogen was used as drying gas at a flow rate of 13 l-min^-1^ and a temperature of 350°C. Nebulizer pressure was set at 60 psi. For quantification purposes, we monitored D-meth in selected-ion monitoring mode (*m/z* = 150).

**Ceramide synthase activity**

Ceramide synthase activity was measured as described [50]. Fresh tissues were collected in homogenization buffer (25 mM HEPES, pH 7.4, containing 5 mM EGTA, 50 mM NaF, and complete mini EDTA-free protease inhibitor; 1 ml). Tissues were disrupted using a pulse homogenizer and centrifuged at 800xg for 5 min. The post nuclear supernatant was centrifuged at 250,000xg for 30 min at 4°C. The microsomal membrane pellet was suspended in homogenization buffer (0.25-0.5 ml). Protein concentration was measured using the BCA protein assay (Pierce). Ceramide synthase activity was determined at 37°C for 1 h in HEPES buffer (20 mM, pH 7.4) containing 2 mM MgCl_2_, fatty acid-free bovine serum albumin (20 µmol), membrane protein (0.05-0.1 mg), using dihydrosphingosine (sphinganine d17:0, 20 µmol) and palmitoyl-coenzyme A (70 µmol) as substrates. The reactions were stopped by adding chloroform-methanol (2:1, v/v) containing ceramide (d18:1/12:0) as internal standard. Lipid extracts were dried under nitrogen and reconstituted in chloroform-methanol (1:3, v/v; 0.1 ml) for LC-MS analyses. Reaction products were measured using an Agilent 1100-LC system coupled to ion-trap XCT and interfaced with ESI (Agilent Technologies). The mobile phase A was methanol containing 0.25% acetic acid and 5 mM ammonium acetate; mobile phase B was water containing 0.25% acetic acid and 5 mM ammonium acetate. Lipids were separated using a reversed-phase Poroshell 300SB C-18 column (2.1x75 mm i.d., coating layer of 0.25 μm on total particle diameter of 5 μm, 300 Å of porous diameter, Agilent Technologies) and identified based on their retention times. A linear gradient was applied from 50% A to 100% B in 6 min at a flow rate of 1.0 ml-min^-1^ with column temperature set at 50°C. The capillary voltage was set at 4.5 kV and skimmer voltage at 40V. Nitrogen was used as drying gas at a flow rate of 10 l-min^-1^, temperature at 350°C and nebulizer pressure at 60 psi. Helium was used as collision gas. For quantification purposes, we monitored the ions at m/z 526.5>508.5 for dihydroceramide (d17:0/16:0) and m/z 482.5>464.5>264.3 for ceramide (d18:1/12:0).

**Senescence and cell toxicity assays**

*Senescence-associated β-Gal staining* was performed as previously reported[[51]](#_ENREF_50). Briefly, we plated MEF on Lab-Tek chamber slides at a density of 5x10^4^ cells per chamber. On the following day, cells were treated with drugs or vehicle for 48 h, cells were washed twice with PBS and fixed in 2% formaldehyde/0.2% glutaraldehyde for 5 min at room temperature. After two PBS washes the slides were incubated with fresh β-galactosidase stain solution [1 mg-ml^-1^ 5-bromo-4-chloro-3-indoyl β-D-galactoside (X-Gal), 40 mM sodium phosphate, pH 6.0, 5 mM potassium ferrocyanide, 5 mM potassium ferricyanide, 150 mM NaCl, and 2 mM MgCl_2_] at 37°C for 12-16 h. Slides were washed with PBS and mounted with 4’6-diamidino-2-phenylindole (DAPI) containing media. The percentage of SA-β-gal positive cells was determined by counting the number of blue cells under bright field illumination, and then the total number of cells in the same filed under fluorescent microscopy (Nikon Eclipse E600). More than 200 cells from 5 different regions of each slide were counted by an observer blinded to experimental condition.

*DNA replication assay.* Cells were seeded in 12-well plates (5x10^4^ per well) and treated with 1 mM D-meth for 48 h. The medium was then replaced with fresh media containing 2.5 µCi/mL of [^3^H] thymidine (6.7 Ci-mmol^-1^, MPBio). After 24 h, cells were rinsed twice with ice-cold PBS and genomic DNA was isolated using DNeasy kits (Qiagen). Radioactivity in genomic DNA was measured by liquid scintillation counting. *Crystal Violet Staining.* Cells were fixed with 4% paraformaldehyde cells and stained with crystal violet (0.5% in methanol/PBS, 1/1). After thoroughly washing with water, images were taken using a Westover Scientific Series 8 microscope.

*Population Doublings.* We plated 1x10^6^ cells on 60 mm dishes and treated them with vehicle or D-meth (1 mM). Cells were trypsinized and re-plated at the same density every 3 days for 6 passages. Population doublings were calculated according to the formula log (final cell number/plated cell number/log2).

*Cell toxicity.* Cells were counted using a hemocytometer and plated at a density of approximately 1x10^3^ cells per well in 96-well microtiter plates in DMEM medium supplemented with 10% FBS and 1% penicillin/streptomycin containing D-meth in a final volume of 0.2 ml. MTT (3-(4,5-dimethylthiazol-2-yl)-2,5-diphenyltetrazolium bromide) and LDH (lactate dehydrogenase) assays were used to measure cell viability. After 48 h, the medium was removed and cells were supplied with fresh medium containing MTT (0.45 mg-ml^-1^). The cells were incubated at 37°C for an additional 3 h, after which the medium was removed; the cells were washed once with PBS, and a solution of 50% dimethylformamide, 10% sodium dodecyl sulfate (DMF/SDS) was added to dissolve the MTT precipitate. The concentrations of MTT and LDH release into the media were measured at 570 nm and 490 nm, respectively, using a SpectraMax plate reader (Molecular Devices, Sunnyvale, CA, USA). Results are presented as relative cell viability compared to control groups.

**Gene expression and silencing**

Total RNA was extracted from frozen tissues using TRIzol reagent (Invitrogen, Carlsbad, CA) and was purified with RNeasy mini kits (Qiagen, Valencia, CA). First-strand complementary DNAs were synthesized using SuperScript II RNaseH reverse transcriptase (Invitrogen). Reverse transcription of total RNA (2 µg) was conducted using oligo(dT)12–18 primers for 50 min at 42°C. mRNA levels were measured by quantitative real-time polymerase chain reaction (RT-PCR) with a Mx 3000P system (Stratagene, La Jolla, CA). The following primers and fluorogenic probes were purchased from Applied Biosystems (TaqMan Gene Expression Assays, Foster City, CA): Ceramide Synthase 1 (Rn01420081_m1), Ceramide Synthase 2 (Rn01762789_m1), Ceramide Synthase 4 (Rn01767402_m1), Ceramide Synthase 5 (Rn01532864_m1), Ceramide Synthase 6 (Rn01270930_m1), Interleukin-6 (Rn01410330_m1). mRNA levels were normalized using β-actin, 18S ribosomal protein or glyceraldehyde-3-phosphate dehydrogenase as internal standards. Additional PCR primers, designed using Primer 3 (https://www. Frodo.wit.mit.edu), are described in Table S8.

Cultures of immortalized MEF were reverse transfected with pooled siRNA sequences against p65 or non-targeting pool as a negative control. Both siRNA pools were purchased from Thermo Scientific (USA). Silencing sequences for p65 were: CCA GAC CGA AGU AUC CAUA; GGG AUG AGA UCU UCU UGC U; GGC AUG CGA UUC CGC UAU A; GCU CAA GAU CUG CCG AGU A. For reverse transfection, 300.000 cells were transfected in 2 ml of Optimem containing 25 mM siRNA and transfection reagent (Dharmafect 1, Thermo scientific, 3 ml). 48 h after transfection, the cells were exposed to vehicle or 1 mM D-Meth for 24 h.

**Chromatin immunoprecipitation (ChIP)**

We used the two-step cross-linking method described in [52]. Briefly, MEFs were grown in DMEM medium supplemented with 10% FBS and 1% penicillin/streptomycin. At confluence, MEFs were treated with 1 mM D-meth or vehicle for 24 h and then processed for two-step cross-linking. Cells were first fixed with disuccinimidyl glutarate (DSG) for 45 min at room temperature. At the end of fixation, the cells were washed 3 times with PBS and then fixed with a freshly prepared solution of 1% (v/v) formaldehyde in PBS for 15 min. After this second cross-link, cells were processed for ChIP using a modified version of the fast ChIP method described in (58). Cells were scraped in IP buffer [150 mM NaCl, 5 mM EDTA, 1% Triton X-100, 0.5% NP-40, 50 mM Tris–HCl (pH 7.5) and 0.5 mM DTT] containing the protease inhibitor cocktail (Sigma). After sonication, shared chromatin was immunoprecipitated overnight at 4°C with an anti-NF-κB p65 antibody (Santa Cruz). Rabbit IgG was used as negative control. Protein A beads were then added and the slurry was rotated at 4°C for 2 hrs. After 6 washes with IP buffer, a total of 100 ml of 10% Chelex (10 g/100 ml H2O) was added directly to the washed protein A beads and vortexed. After 10 min boiling, the Chelex/protein A bead suspension was allowed to cool to room temperature. Proteinase K (100 mg/ml) was added and beads were incubated for 30 min at 55°C while shaking, followed by another round of boiling for 10 min. The suspension was centrifuged and supernatant was collected. The Chelex/protein A beads fraction was vortexed with another 100 ml water, centrifuged again, and the first and the second supernatants were combined. Eluate was used directly as a template in real-time PCR and made up to 25% of the final reaction volume. Results are reported as percentage of input (%) calculated according to the formula: 100 x 2^(Ctadjusted Input – CtEnriched). Input DNA Ct was adjusted from 1% to 100% equivalent by subtracting 6.644 Cts or Log2 100.

**Statistical analyses**

Lipid-level data from the self-administration experiment were logarithmically transformed and analyzed using restricted maximum likelihood estimation (Proc Mixed; SAS Institute, Cary, NC; see Littel, RC, Milliken GA, Stroup WW, Wolfinger RD, Schabenberger, O (2006) SAS for Mixed Models, 2nd edn. SAS Institute, Cary, NC). Each lipid family was analyzed separately for brain and peripheral tissues, with D-meth exposure as a between-subjects factor and lipid species as a within-subjects factor. Residuals under this mixed model were normally distributed. P values from Proc Mixed were used to perform planned comparisons between the D-meth and control group for each lipid species or family; to correct for multiple comparisons, an overall false discovery rate [53] of 0.05 was maintained for the entire experiment, comprising all data in Figs. 1a, 1b, and 1c (and the corresponding Tables S1, S2, S3, and S4). For graphic presentation of group results, heat maps were generated using the Studentized value for each comparison, such that each cell represents the size of the difference between the means of the D-meth and control groups, divided by the pooled standard error; red cells indicate increased lipid levels in the D-meth group, and green cells indicate decreased levels. For graphic presentation of individual-subject results, heat maps were generated by normalizing the data for each lipid species relative to the mean and standard error of the control group,; the brightness of the color indicates the number of standard errors above (red cells) or below (green cells) the mean of the control group. For the remaining data sets (i.e., all data except those shown in Figures 1a, 1b, and 1c and the corresponding Tables S1, S2, S3, and S4), comparisons of parameters were made by Student’s t test when tere were two groups and by one-way analysis of variance when there were more than two groups; comparisons of different parameters between each group were made by a post hoc analysis using a Bonferroni test. Statistical significance was evaluated with GraphPad Prism5. A value of P < 0.05 was considered to be statistically significant. Descriptive statistics are presented as means ± SD.
