# Supplementary material for: Methamphetamine Accelerates Cellular Senescence through Stimulation of De Novo Ceramide Biosynthesis
Source: PLoS One. 2015 Feb 11;10(2):e0116961. doi: 10.1371/journal.pone.0116961 (PMC4324822; doi:10.1371/journal.pone.0116961)
Supplement: S1 Table — Abbreviations: DAG, diacylglycerol; DHC, dihydro-ceramide; DH-Sphm, dihydro-sphingomyelin; MAG, monoacylglycerol; M-FA, monounsaturated fatty acid; PC, phosphatidylcholine; PE, phosphatidylethanolamine; P-FA, polyunsaturated fatty acid; PG, phosphatidylglycerol; PI, phosphatidylinositol; PS, phosphatidylserine; S-FA, saturated fatty acid; Sphm, sphingomyelin; TAG, triacylglycerol. Values are expressed as mean±s.e.m. *P<0.05, P; ***P<0.001; N.D., not detected; planned comparisons obtained from Proc Mixed analysis with False Discovery Rate correction for multiple comparisons (n = 12 in D-meth group and 6 in control group). (DOCX) [file pone.0116961.s012.docx]

**Table S1:** **Levels of various lipid species in brain regions of rats self-administering D-meth and yoked control rats.** Abbreviations: DAG, diacylglycerol; DHC, dihydro-ceramide; DH-Sphm, dihydro-sphingomyelin; MAG, monoacylglycerol; M-FA, monounsaturated fatty acid; PC, phosphatidylcholine; PE, phosphatidylethanolamine; P-FA, polyunsaturated fatty acid; PG, phosphatidylglycerol; PI, phosphatidylinositol; PS, phosphatidylserine; S-FA, saturated fatty acid; Sphm, sphingomyelin; TAG, triacylglycerol. Values are expressed as mean±s.e.m. *P<0.05, P; ***P<0.001; N.D., non detected; planned comparisons obtained from Proc Mixed analysis with False Discovery Rate correction for multiple comparisons (n = 12 in D-meth group and 6 in control group).

| Lipid species | Condition | Frontal Cortex | Dorsal Striatum | Ventral Striatum | Hippocampus | Cerebellum |
| --- | --- | --- | --- | --- | --- | --- |
| Ceramide (nmol/g) | Control | 4.64 ± 0.07 * | 4.09 ± 0.07 *** | 4.33 ± 0.05 * | 4.17 ± 0.06 | 3.7 ± 0.12 |
|  | Meth | 4.83 ± 0.03 | 4.6 ± 0.04 | 4.49 ± 0.03 | 4.12 ± 0.07 | 3.78 ± 0.08 |
| DH-Cer (nmol/g) | Control | 6.49 ± 0.47 * | 5.8 ± 0.47 * | 5.34 ± 0.31 | 4.34 ± 0.57 | 7.09 ± 1.23 |
|  | Meth | 8.4 ± 0.42 | 9.45 ± 0.65 | 7.11 ± 0.46 | 3.97 ± 0.25 | 9.09 ± 1.02 |
| Sphm (nmol/mg) | Control | 2.18 ± 0.21 | 2.35 ± 0.21 | 2.29 ± 0.14 | 1.94 ± 0.09 | 2.45 ± 0.17 |
|  | Meth | 2.12 ± 0.17 | 2.42 ± 0.09 | 2.35 ± 0.08 | 2.07 ± 0.08 | 3.01 ± 0.2 |
| DH-Sphm (nmol/mg) | Control | 0.35 ± 0.05 | 0.37 ± 0.05 | 0.31 ± 0.02 | 0.26 ± 0.02 | 0.51 ± 0.04 |
|  | Meth | 0.27 ± 0.02 | 0.37 ± 0.02 | 0.3 ± 0.02 | 0.25 ± 0.01 | 0.64 ± 0.05 |
| S-FA (nmol/g) | Control | 1833.85 ± 117.06 * | 1626.76 ± 117.06 | 1532.63 ± 132.2 | 1984.53 ± 125.15 | 2285.94 ± 118.43 |
|  | Meth | 2218.18 ± 92.37 | 1617.54 ± 53.78 | 1735.4 ± 75.76 | 1797.04 ± 161.7 | 2315.5 ± 90.31 |
| M-FA (nmol/g) | Control | 342.75 ± 21.81 | 189.48 ± 21.81 | 239.69 ± 6.93 | 291.83 ± 11.05 | 213.51 ± 15.39 |
|  | Meth | 359.89 ± 14.91 | 187.68 ± 7.03 | 235 ± 11.33 | 303.42 ± 15.03 | 255.59 ± 15.07 |
| P-FA (nmol/g) | Control | 618.14 ± 45.4 | 432.52 ± 45.4 | 605.03 ± 30.68 | 613.35 ± 22.69 | 311.13 ± 29.58 |
|  | Meth | 652.46 ± 19.81 | 489.15 ± 19.7 | 590.55 ± 26.63 | 597.19 ± 22.7 | 365.54 ± 21.39 |
| MAG (nmol/g) | Control | 943.46 ± 48.45 | 468.16 ± 48.45 | 561.72 ± 20.08 | 871.53 ± 28.84 | 405.02 ± 25.03 |
|  | Meth | 1048.92 ± 42.43 | 509.76 ± 16.78 | 602.81 ± 22.49 | 949.53 ± 26.58 | 460.21 ± 15.65 |
| DAG (nmol/mg) | Control | 4.88 ± 0.67 | 0.55 ± 0.06 | 0.43 ± 0.03 | 0.48 ± 0.04 | 0.541 ± 0.03 |
|  | Meth | 4.61 ± 0.32 | 0.59 ± 0.03 | 0.49 ± 0.03 | 0.52 ± 0.02 | 0.40 ± 0.04 |
| TAG (nmol/mg) | Control | 19.39 ± 1.0 | 11.65 ± 1.05 | 17.76 ± 0.56 * | 10.83 ± 0.41 | 23.68 ± 3.79 |
|  | Meth | 19.60 ± 0.86 | 11.07 ± 0.42 | 20.96 ± 0.99 | 10.92 ± 0.36 | 23.42 ± 2.4 |
| PE (nmol/mg) | Control | 32.48 ± 5.02 | 36.58 ± 5.02 | 31.32 ± 2.96* | 25.2 ± 2.17 | 26.62 ± 1.65 |
|  | Meth | 34.43 ± 1.95 | 38.63 ± 2.25 | 24.11 ± 1.35 | 24.01 ± 1.09 | 32.99 ± 1.87 |
| PC (nmol/mg) | Control | 8.35 ± 1.01 | 8.87 ± 1.01 | 9.13 ± 0.36 | 9.37 ± 0.59 | 9.04 ± 0.37 |
|  | Meth | 8.27 ± 0.51 | 9.26 ± 0.33 | 9.01 ± 0.27 | 9.01 ± 0.33 | 9.76 ± 0.35 |
| PS (nmol/mg) | Control | 24.03 ± 4.05 | 50.05 ± 4.05 * | 15.31 ± 1.24 | 15.38 ± 1.96 | 49.46 ± 8.83 |
|  | Meth | 23.54 ± 1.73 | 22.89 ± 4.36 | 11.79 ± 1 | 12.2 ± 0.72 | 56.33 ± 5.46 |
| PI (nmol/mg) | Control | 24.42 ± 4.75 | 25.49 ± 4.75 | 19.91 ± 1.75 * | 12.17 ± 1.94 | 29.96 ± 4.08 |
|  | Meth | 19.12 ± 2.12 | 22.58 ± 1.13 | 14.48 ± 0.72 | 9.56 ± 0.69 | 36.38 ± 3.74 |
| PG (nmol/mg) | Control | 0.65 ± 0.05 | 1.85 ± 0.05 | 1.76 ± 0.04 * | 2.42 ± 0.4 | 1.77 ± 0.1 |
|  | Meth | 0.73 ± 0.05 | 1.99 ± 0.07 | 2.68 ± 0.26 | 2.53 ± 0.19 | 2.15 ± 0.16 |
