# Supplementary material for: Methamphetamine Accelerates Cellular Senescence through Stimulation of De Novo Ceramide Biosynthesis
Source: PLoS One. 2015 Feb 11;10(2):e0116961. doi: 10.1371/journal.pone.0116961 (PMC4324822; doi:10.1371/journal.pone.0116961)
Supplement: S2 Table — Values are expressed as mean±s.e.m. *P<0.05, P; N.D., not detected; planned comparisons obtained from Proc Mixed analysis with False Discovery Rate correction for multiple comparisons (n = 6 in D-meth group and 6 in control group). (DOCX) [file pone.0116961.s013.docx]

| Ceramide | Condition | | Frontal Cortex | | Dorsal Striatum | | Ventral Striatum | | Hippocampus | | Cerebellum | |
| --- | --- | --- | --- | --- | --- | --- | --- | --- | --- | --- | --- | --- |
| (d18:1/14:0) | | Control | | 0.08 ± 0.01 | | 0.04 ± 0.00 | | 0.05 ± 0.01 | | 0.05 ± 0.01 | | 1.16 ± 0.57 |
|  |  | Meth | | 0.07 ± 0.01 | | 0.04 ± 0.00 | | 0.04 ± 0.00 | | 0.04 ± 0.00 | | 1.32 ± 0.43 |
|  |  |  | |  | |  | |  | |  | |  |
| (d18:1/16:0) | | Control | | 0.94 ± 0.12 | | 0.56± 0.02* | | 0.64 ± 0.03 | | 0.55 ± 0.02 | | 1.05 ± 0.57 |
|  |  | Meth | | 1.01 ± 0.07 | | 0.78 ± 0.03 | | 0.66 ± 0.02 | | 0.52 ± 0.02 | | 3.04 ± 1.44 |
|  |  |  | |  | |  | |  | |  | |  |
| (d18:1/18:0) | | Control | | 102.62 ± 6.86* | | 58.96 ± 4.59* | | 74.47 ± 3.44* | | 63.58 ± 3.87 | | 14.48 ± 4.06 |
|  |  | Meth | | 124.05 ± 5.13 | | 98.11 ± 4.07 | | 87.93 ± 2.79 | | 61.86 ± 4.06 | | 21.15 ± 3.25 |
|  |  |  | |  | |  | |  | |  | |  |
| (d18:1/24:1) | | Control | | 0.62 ± 0.15 | | 0.35 ± 0.08 | | 0.60 ± 0.11 | | 0.32 ± 0.06 | | 233.59 ± 43.04 |
|  |  | Meth | | 0.72 ± 0.11 | | 0.76 ± 0.17 | | 0.44 ± 0.04 | | 0.49 ± 0.10 | | 434.96 ± 123.84 |
|  |  |  | |  | |  | |  | |  | |  |
| (d18:1/24:0) | | Control | | 0.51 ± 0.03* | | 0.41 ± 0.03* | | 0.48 ± 0.03* | | 0.46 ± 0.02 | | N.D. |
|  |  | Meth | | 0.68 ± 0.04 | | 0.65 ± 0.30 | | 0.64 ± 0.05 | | 0.46 ± 0.03 | | N.D. |
|  |  |  | |  | |  | |  | |  | |  |
| (d18:1/26:0) | | Control | | 0.06 ± 0.01 | | 0.05 ± 0.01 | | 0.03 ± 0.00 | | 0.03 ± 0.00 | | N.D. |
|  |  | Meth | | 0.08 ± 0.01 | | 0.05 ± 0.00 | | 0.03 ± 0.00 | | 0.03 ± 0.00 | | N.D. |

**Table S2:** Levels of ceramide species in brain regions of rats self-administering D-meth and yoked control rats. Values are expressed as mean±s.e.m. *P<0.05, P; N.D., non detected; planned comparisons obtained from Proc Mixed analysis with False Discovery Rate correction for multiple comparisons (n=6 in D-meth group and 6 in control group).
