# Supplementary material for: Methamphetamine Accelerates Cellular Senescence through Stimulation of De Novo Ceramide Biosynthesis
Source: PLoS One. 2015 Feb 11;10(2):e0116961. doi: 10.1371/journal.pone.0116961 (PMC4324822; doi:10.1371/journal.pone.0116961)
Supplement: S3 Table — Abbreviations: DAG, diacylglycerol; DHC, dihydroceramide; DH-Sphm, dihydro-sphingomyelin; MAG, monoacylglycerol; M-FA, monounsaturated fatty acid; PC, phosphatidylcholine; PE, phosphatidylethanolamine; P-FA, polyunsaturated fatty acid; PG, phosphatidylglycerol; PI, phosphatidylinositol; PS, phosphatidylserine; S-FA, saturated fatty acid; Sphm, sphingomyelin; TAG, triacylglycerol. Values are expressed as mean±s.e.m. *P<0.05; N.D., not detected; planned comparisons obtained from Proc Mixed analysis with False Discovery Rate correction for multiple comparisons (n = 6 in D-meth group and 6 in control group). (DOCX) [file pone.0116961.s014.docx]

**Table S3:** Levels of various lipid species in organs and tissues of rats self-administering D-meth and yoked control rats. Abbreviations: DAG, diacylglycerol; DHC, dihydroceramide; DH-Sphm, dihydro-sphingomyelin; MAG, monoacylglycerol; M-FA, monounsaturated fatty acid; PC, phosphatidylcholine; PE, phosphatidylethanolamine; P-FA, polyunsaturated fatty acid; PG, phosphatidylglycerol; PI, phosphatidylinositol; PS, phosphatidylserine; S-FA, saturated fatty acid; Sphm, sphingomyelin; TAG, triacylglycerol. Values are expressed as mean±s.e.m. *P<0.05; N.D., non detected; planned comparisons obtained from Proc Mixed analysis with False Discovery Rate correction for multiple comparisons (n = 6 in D-meth group and 6 in control group).

| Lipid species | Condition | Sk.Muscle | Heart | Liver | Kidney | Skin | Spleen | Pancreas |
| --- | --- | --- | --- | --- | --- | --- | --- | --- |
| Ceramide (nmol/g) | Control | 23.63 ± 1.13* | 10.92 ± 0.67* | 47.29 ± 2.55* | 8.64 ± 0.85* | 52.67 ± 4.88* | 35.42 ± 1.59* | 81.54 ± 2.2 |
|  | Meth | 42.61 ± 2.4 | 17.14 ± 1.35 | 74.73 ± 6.03 | 12.42 ± 0.74 | 67.73 ± 2.87 | 42.01 ± 1.09 | 70.39 ± 7.49 |
| DH-Cer (nmol/g) | Control | 12.86 ± 1.32* | 14.56 ± 1.14 | 170.21 ± 12.87 | 8.21 ± 0.7 | 25.01 ± 4.87* | 42.73 ± 2.16 | 92.63 ± 4.91 |
|  | Meth | 19.31 ± 1.45 | 17.18 ± 0.91 | 151.03 ± 5.78 | 8.99 ± 0.39 | 41.67 ± 3.04 | 37.81 ± 2.38 | 126.51 ± 12.68 |
| Sphm (nmol/mg) | Control | 0.69 ± 0.04 | 2.37 ± 0.27 | 3.31 ± 0.23 | 4.57 ± 0.35 | 1.62 ± 0.11 | 1.76 ± 0.05 | 1.85 ± 0.15 |
|  | Meth | 0.77 ± 0.04 | 2.66 ± 0.19 | 3.5 ± 0.28 | 4.22 ± 0.22 | 1.73 ± 0.06 | 1.71 ± 0.07 | 1.46 ± 0.11 |
| DH-Sphm (nmol/mg) | Control | 2.52 ± 0.15 | 3.19 ± 0.16 | 2.65 ± 0.07 | 5.88 ± 0.47 | 6.13 ± 0.86 | 12.44 ± 1.02 | 6.17 ± 0.72* |
|  | Meth | 3.01 ± 0.23 | 3.15 ± 0.18 | 3.28 ± 0.23 | 6.38 ± 0.52 | 4.98 ± 0.3 | 12.22 ± 1 | 11.56 ± 1.1 |
| S-FA (nmol/g) | Control | 414.14 ± 148.58 | 530.15 ± 37.8 | 802.84 ± 61.7* | 630.46 ± 43.14 | 1913.57 ± 99.9 | 861.19 ± 55.4 | 2962.21 ± 479.14 |
|  | Meth | 1901.91 ± 296.56 | 491.98 ± 13.39 | 616.69 ± 26.78 | 707.21 ± 36.12 | 1668.47 ± 64.92 | 664.87 ± 24.15 | 2431.9 ± 249.5 |
| MAG (nmol/g) | Control | 87.72 ± 18.75 | 186.84 ± 9.93 | 208.04 ± 14.24* | 201.42 ± 14.51 | 145.95 ± 6.62 | 364.39 ± 34.81 | 1390.31 ± 37.89 |
|  | Meth | 152.76 ± 22.88 | 202.19 ± 11.16 | 175.25 ± 4.5 | 211.07 ± 13.34 | 138.05 ± 4.55 | 284.02 ± 11.68 | 1247.96 ± 150.15 |
| DAG (nmol/mg) | Control | 10.84 ± 4.09 | 110.38 ± 10.75 | 290444.39 ± 51953.56 | 152.85 ± 18.48 | 160.75 ± 9.43 | 5.68 ± 0.23 | 25.45 ± 1.98 |
|  | Meth | 6.92 ± 1.02 | 142.67 ± 11.2 | 315424.17 ± 23365.74 | 275.78 ± 61.2 | 147.77 ± 6.07 | 5.96 ± 0.47 | 46.04 ± 4.67 |
| TAG (nmol/mg) | Control | 21.89 ± 9.3 | 23.17 ± 2.32 | 24.97 ± 2.06 | 27.02 ± 3.13 | 28.05 ± 4.97 | 17.24 ± 1.48 | 90.4 ± 28.55 |
|  | Meth | 14.24 ± 1.03 | 19.76 ± 1.36 | 24.57 ± 1.9 | 26.34 ± 3.18 | 13.29 ± 1.68 | 14.75 ± 0.99 | 172.43 ± 57.44 |
| PE (nmol/mg) | Control | 4.18 ± 0.36 | 6.19 ± 0.48 | 4.62 ± 0.32 | 7.24 ± 0.69 | 2.44 ± 0.31 | 17.01 ± 0.8 | 14.23 ± 1.65 |
|  | Meth | 5.29 ± 0.35 | 6.92 ± 0.33 | 4.79 ± 0.21 | 6.67 ± 0.4 | 2.23 ± 0.14 | 12.63 ± 0.88 | 19.23 ± 1.72 |
| PC (nmol/mg) | Control | 2.91 ± 0.19 | 4.19 ± 0.23 | 5.63 ± 0.25* | 6 ± 0.31 | 2.51 ± 0.16 | 3.39 ± 0.07 | 4.61 ± 0.19 |
|  | Meth | 3.13 ± 0.1 | 4.31 ± 0.12 | 4.3 ± 0.12 | 5.45 ± 0.16 | 2.33 ± 0.07 | 3.35 ± 0.12 | 3.68 ± 0.4 |
| PS (nmol/mg) | Control | 2.06 ± 0.17 | 2.79 ± 0.13 | 2.28 ± 0.19 | 3.63 ± 0.32 | 1.65 ± 0.28 | 6.1 ± 0.27 | 5.57 ± 0.59 |
|  | Meth | 2.36 ± 0.16 | 2.99 ± 0.18 | 2.34 ± 0.13 | 3.54 ± 0.23 | 1.24 ± 0.09 | 5.19 ± 0.35 | 7.57 ± 0.8 |
| PI (nmol/mg) | Control | 1.65 ± 0.24 | 1.52 ± 0.25 | 4.92 ± 0.4 | 7.72 ± 0.54 | 2.04 ± 0.89 | 8.28 ± 0.4 | 13.02 ± 1.04 |
|  | Meth | 1.52 ± 0.13 | 1.91 ± 0.13 | 4.85 ± 0.24 | 8.33 ± 0.51 | 1.24 ± 0.07 | 7.67 ± 0.7 | 15.17 ± 1.92 |
| PG (nmol/mg) | Control | 0.8 ± 0.1 | 2.67 ± 0.26 | 1.6 ± 0.12 | 2.75 ± 0.14 | 1.83 ± 0.18 | 0.94 ± 0.07 | 0.43 ± 0.03 |
|  | Meth | 0.98 ± 0.07 | 2.97 ± 0.14 | 1.71 ± 0.08 | 3.11 ± 0.2 | 1.61 ± 0.08 | 0.96 ± 0.05 | 0.4 ± 0.03 |
