# Supplementary material for: Methamphetamine Accelerates Cellular Senescence through Stimulation of De Novo Ceramide Biosynthesis
Source: PLoS One. 2015 Feb 11;10(2):e0116961. doi: 10.1371/journal.pone.0116961 (PMC4324822; doi:10.1371/journal.pone.0116961)
Supplement: S4 Table — Values are expressed as mean±s.e.m. *P<0.05, **P<0.01; ***P<0.001; N.D., not detected; planned comparisons obtained from Proc Mixed analysis with False Discovery Rate correction for multiple comparisons; (n = 6 in D-meth group and 6 in control group). (DOCX) [file pone.0116961.s015.docx]

**Table S4:** Levels of ceramide species in peripheral tissues of rats self-administering D-meth and yoked control rats. Values are expressed as mean±s.e.m. *P<0.05, **P<0.01; ***P<0.001; N.D., non detected; planned comparisons obtained from Proc Mixed analysis with False Discovery Rate correction for multiple comparisons; (n = 6 in D-meth group and 6 in control group).

| Ceramide | Condition | | Skeletal Muscle | | | | Heart | | | | Liver | | | | | Kidney | | | | Skin | | | | Spleen | | | | Pancreas | | |
| --- | --- | --- | --- | --- | --- | --- | --- | --- | --- | --- | --- | --- | --- | --- | --- | --- | --- | --- | --- | --- | --- | --- | --- | --- | --- | --- | --- | --- | --- | --- |
| (d18:1/14:0) | | Control | | 0.01 ± 0.00 | | | | 0.005 ± 0.00 | | | | 0.03 ± 0.00* | | | | | 0.03 ± 0.01 | | | | 0.83 ± 0.15* | | | | 0.01 ± 0.00 | | | | 0.07 ± 0.01** | |
|  |  | Meth | | 0.01 ± 0.00 | | | | 0.006 ± 0.00 | | | | 0.018 ± 0.00 | | | | | 0.04 ± 0.01 | | | | 1.27 ± 0.08 | | | | 0.01 ± 0.00 | | | | 0.03 ± 0.00 | |
|  |  |  | |  | | | |  | | | |  | | | | |  | | | |  | | | |  | | | |  | |
| (d18:1/16:0) | | Control | | 0.76 ± 0.13*** | | | | 1.05 ± 0.18** | | | | 9.55 ± 1.01* | | | | | 6.84 ± 0.98* | | | | 40.49 ± 3.99 | | | | 9.68 ± 0.88* | | | | 19.91 ± 1.33 | |
|  |  | Meth | | 4.02 ± 0.33 | | | | 1.7 ± 0.15 | | | | 15.37 ± 1.44 | | | | | 9.76 ± 0.57 | | | | 47.04 ± 1.79 | | | | 11.97 ± 0.55 | | | | 20.01 ± 1.29 | |
|  |  |  | |  | | | |  | | | |  | | | | |  | | | |  | | | |  | | | |  | |
| (d18:1/18:0) | | Control | | 15.33 ± 1.17*** | | | | 1.92 ± 0.14** | | | | 0.7 ± 0.1** | | | | | 0.01 ± 0.00 | | | | 3.84 ± 0.78** | | | | 2.52 ± 0.32 | | | | 2.89 ± 0.55 | |
|  |  | Meth | | 25.55 ± 1.68 | | | | 4.01 ± 0.51 | | | | 2.03 ± 0.27 | | | | | 0.01 ± 0.00 | | | | 5.88 ± 0.3 | | | | 3.03 ± 0.16 | | | | 2.16 ± 0.27 | |
|  |  |  | |  | | | |  | | | |  | | | | |  | | | |  | | | |  | | | |  | |
| (d18:1/24:1) | | Control | | 1.34 ± 0.19*** | | | | 0.73 ± 0.05*** | | | | 8.14 ± 0.85 | | | | | 0.04 ± 0.01 | | | | 0.56 ± 0.1 | | | | 8.39 ± 0.56** | | | | 16.64 ± 1.45 | |
|  |  | Meth | | 3.51 ± 0.33 | | | | 1.88 ± 0.22 | | | | 10.95 ± 1.51 | | | | | 0.06 ± 0.00 | | | | 0.82 ± 0.11 | | | | 10.34 ± 0.32 | | | | 11.57 ± 1.68 | |
|  |  |  | |  | | | |  | | | |  | | | | |  | | | |  | | | |  | | | |  | |
| (d18:1/24:0) | | Control | | 6.27 ± 0.57* | | | | 7.13 ± 0.43 | | | | 28.16 ± 1.94 | | | | | 1.60 ± 0.07* | | | | 6.38 ± 1.03** | | | | 14.55 ± 0.71 | | | | 40.79 ± 2.37 | |
|  |  | Meth | | 9.44 ± 0.77 | | | | 9.43 ± 0.65 | | | | 45.58 ± 5.01 | | | | | 2.52 ± 0.22 | | | | 11.77 ± 1.02 | | | | 16.43 ± 0.85 | | | | 35.55 ± 4.29 | |
|  |  |  | |  | | | |  | | | |  | | | | |  | | | |  | | | |  | | | |  | |
| (d18:1/26:0) | | Control | | 0.04 ± 0.01 | | | | 0.09 ± 0.01 | | | | 0.85 ± 0.04 | | | | | 0.03 ± 0.01 | | | | 0.58 ± 0.12 | | | | 0.26 ± 0.03 | | | | 1.24 ± 0.25 | |
|  |  | Meth | | 0.07 ± 0.01 | | | | 0.1 ± 0.01 | | | | 0.78 ± 0.09 | | | | | 0.03 ± 0.00 | | | | 0.95 ± 0.12 | | | | 0.22 ± 0.02 | | | | 1.06 ± 0.29 | |
|  |  |  | | | |  | | | |  | | | |  | | | |  | | | |  | | | |  | | | |  |
|  | |  | | |  | | | |  | | | |  | |  | | | |  | | | |  | | | |  | | | |
