# Supplementary material for: Methamphetamine Accelerates Cellular Senescence through Stimulation of De Novo Ceramide Biosynthesis
Source: PLoS One. 2015 Feb 11;10(2):e0116961. doi: 10.1371/journal.pone.0116961 (PMC4324822; doi:10.1371/journal.pone.0116961)
Supplement: S5 Table — Values are expressed as mean±s.e.m. of mRNA/GAPDH*1000. *P<0.05, P; **P<0.01; ***P<0.001; N.D., not detected; two-tailed Student’s t test (n = 6–12). (DOCX) [file pone.0116961.s016.docx]

**Table S5:** Levels of mRNAs encoding for enzymes of *de novo* ceramide biosynthesis in brain regions of rats self-administering D-meth and yoked control rats. Values are expressed as mean±s.e.m. of mRNA/GAPDH*1000. *P<0.05, P; **P<0.01; ***P<0.001; N.D., non detected; two-tailed Student’s t

test (n = 6-12).

| **Dorsal striatum** |  |  |  |
| --- | --- | --- | --- |
| Gene | Control | Meth | P value |
| Serine palmitoyltransferase 1 | 3.12 ± 0.29 | 3.65 ± 0.26 | 0.204 |
| Serine palmitoyltransferase 2 | 8.61 ± 0.41 | 9.36 ± 0.68 | 0.371 |
| Serine palmitoyltransferase 3 | N.D. | N.D. | N.D. |
| Ceramide Synthase 1 * | 37.46 ± 3.192 | 49.12 ± 3.47 | 0.039 |
| Ceramide Synthase 2 *** | 12.88 ± 0.74 | 7.70 ± 0.239 | 0.001 |
| Ceramide Synthase 4 * | 8.07 ± 0.73 | 11.29 ± 0.93 | 0.028 |
| Ceramide Synthase 5 | 17.20 ± 1.31 | 20.74 1.65 | 0.132 |
| Ceramide Synthase 6 * | 3.73 ± 0.39 | 5.70 ± 0.51 | 0.015 |

| **Frontal cortex** | |  |  |  |
| --- | --- | --- | --- | --- |
| Gene | | Control | Meth | P value |
| Serine palmitoyltransferase 1 | 7.13 ± 0.22 | | 7.10 ± 0.25 | 0.959 |
| Serine palmitoyltransferase 2 | 31.25 ± 1.03 | | 31.08 ± 1.37 | 0.899 |
| Serine palmitoyltransferase 3 | 0.0024± 0.0004 | | 0.0016 ± 0.0004 | 0.343 |
| Ceramide Synthase 1 | 29.55 ± 2.15 | | 28.76 ± 0.86 | 0.686 |
| Ceramide Synthase 2 *** | 18.58 ± 0.48 | | 14.35 ± 0.53 | 0.000 |
| Ceramide Synthase 4 | 4.14 ± 0.37 | | 4.22 ± 0.28 | 0.871 |
| Ceramide Synthase 5 *** | 15.57 ± 0.48 | | 19.44 ± 0.55 | 0.000 |
| Ceramide Synthase 6 ** | 2.80 ± 0.18 | | 4.26 ± 0.25 | 0.002 |

| **Cerebellum** |  |  |  |
| --- | --- | --- | --- |
| Gene | Control | Meth | P value |
| Serine palmitoyltransferase 1 | 1.52 ± 0.35 | 1.45 ± 0.27 | 0.871 |
| Serine palmitoyltransferase 2 | 5.12 ± 0.18 | 5.51 ± 0.24 | 0.302 |
| Serine palmitoyltransferase 3 | N.D. | N.D. | N.D. |
| Ceramide Synthase 1 | 15.58 ± 1.67 | 13.94 ± 0.65 | 0.283 |
| Ceramide Synthase 2 | 5.77 ± 0.99 | 4.973 ± 0.86 | 0.601 |
| Ceramide Synthase 4 | 0.62 ± 0.12 | 0.88 ± 0.09 | 0.109 |
| Ceramide Synthase 5 | 10.46 ± 1.21 | 11.99 ± 0.89 | 0.333 |
| Ceramide Synthase 6 *** | 0.64 ± 0.05 | 1.15 ± 0.05 | 0.001 |
