# Supplementary material for: Methamphetamine Accelerates Cellular Senescence through Stimulation of De Novo Ceramide Biosynthesis
Source: PLoS One. 2015 Feb 11;10(2):e0116961. doi: 10.1371/journal.pone.0116961 (PMC4324822; doi:10.1371/journal.pone.0116961)
Supplement: S6 Table — Values are expressed as mean±s.e.m. of mRNA/GAPDH*1000. *P<0.05, P; **P<0.01; ***P<0.001; N.D., not detected; two-tailed Student’s t test (n = 6–12). (DOCX) [file pone.0116961.s017.docx]

**Table S6:** Levels of mRNAs encoding for enzymes of *de novo* ceramide biosynthesis in peripheral tissues of rats self-administering D-meth and yoked control rats. Values are expressed as mean±s.e.m. of mRNA/GAPDH*1000. *P<0.05, P; **P<0.01; ***P<0.001; N.D., non detected; two-tailed Student’s t test (n= 6-12).

| **Liver** |  |  |  |
| --- | --- | --- | --- |
| Gene | Control | Meth | P value |
| Serine palmitoyltransferase 1 | 2.22 ± 0.54 | 3.71 ± 0.53 | 0.085 |
| Serine palmitoyltransferase 2 * | 17.70 ± 2.85 | 26.12 ± 0.85 | 0.022 |
| Serine palmitoyltransferase 3 | N.D. | N.D. | N.D. |
| Ceramide Synthase 1 | 0.09 ± 0.03 | 0.16 ± 0.12 | 0.066 |
| Ceramide Synthase 2 | 59.24 ± 6.02 | 61.88 ± 8.73 | 0.810 |
| Ceramide Synthase 4 * | 0.11 ± 0.03 | 0.20 ± 0.03 | 0.046 |
| Ceramide Synthase 5 * | 0.0078 ± 0.0012 | 0.0119 ± 0.0011 | 0.035 |
| Ceramide Synthase 6 | 1.98 ± 0.23 | 1.86 ± 0.53 | 0.836 |

| **Skeletal Muscle** |  |  |  |
| --- | --- | --- | --- |
| Gene | C | M | P-value |
| Serine palmitoyltransferase 1 * | 0.45 ± 0.07 | 1.07 ± 0.15 | 0.022 |
| Serine palmitoyltransferase 2 ** | 0.44 ± 0.05 | 2.46 ± 0.33 | 0.002 |
| Serine palmitoyltransferase 3 | N/A | N/A | N/A |
| Ceramide Synthase 1 | 0.95 ± 0.11 | 0.88 ± 0.12 | 0.729 |
| Ceramide Synthase 2 *** | 1.31 ± 0.15 | 4.32 ± 0.35 | 0.0001 |
| Ceramide Synthase 4 *** | 0.09 ± 0.01 | 0.57 ± 0.07 | 0.0004 |
| Ceramide Synthase 5 | 0.14 ± 0.01 | 0.13 ± 0.02 | 0.685 |
| Ceramide Synthase 6 * | 0.004 ± 0.001 | 0.02 ± 0.004 | 0.032 |

| **Heart** |  |  |  |
| --- | --- | --- | --- |
| Gene | Control | Meth | P value |
| Serine palmitoyltransferase 1 *** | 2.25 ± 0.13 | 3.32 ± 0.23 | 0.004 |
| Serine palmitoyltransferase 2 * | 6.98 ± 0.44 | 10.91 ± 1.00 | 0.011 |
| Serine palmitoyltransferase 3 | N.D. | N.D. | N.D. |
| Ceramide Synthase 1 | 0.47 ± 0.07 | 0.54 ± 0.07 | 0.540 |
| Ceramide Synthase 2 | 10.68 ± 0.89 | 12.38 ± 0.62 | 0.130 |
| Ceramide Synthase 4 | 2.51 ± 0.33 | 2.92 ± 0.13 | 0.201 |
| Ceramide Synthase 5 ** | 1.23 ± 0.09 | 1.71 ± 0.08 | 0.002 |
| Ceramide Synthase 6 * | 0.17 ± 0.01 | 0.34 ± 0.05 | 0.013 |

| **Skin** |  |  |  |
| --- | --- | --- | --- |
| Gene | Control | Meth | P value |
| Ceramide Synthase 1 * | 0.34 ± 0.15 | 1.05 ± 0.17 | 0.027 |
| Ceramide Synthase 2 | 15.69 ± 3.94 | 35.11 ± 5.58 | 0.096 |
| Ceramide Synthase 4 * | 1.03 ± 0.40 | 2.73 ± 0.55 | 0.042 |
| Ceramide Synthase 5 * | 0.05 ± 0.01 | 0.50 ± 0.11 | 0.014 |
| Ceramide Synthase 6 | 0.33 ± 0.10 | 0.70 ± 0.14 | 0.132 |
