# Supplementary material for: Methamphetamine Accelerates Cellular Senescence through Stimulation of De Novo Ceramide Biosynthesis
Source: PLoS One. 2015 Feb 11;10(2):e0116961. doi: 10.1371/journal.pone.0116961 (PMC4324822; doi:10.1371/journal.pone.0116961)
Supplement: S7 Table — Values are expressed as mean±s.e.m. of mRNA/GAPDH*1000. *P<0.05, P; **P<0.01; ***P<0.001; two-tailed Student’s t test (n = 6–12). (DOCX) [file pone.0116961.s018.docx]

**Table S7:** Levels of mRNAs encoding for Tumor Necrosis Factor-α (TNF-α) and interleukin-6 in peripheral tissues of rats self-administering D-meth and yoked control rats. Values are expressed as mean±s.e.m. of mRNA/GAPDH*1000. *P<0.05, P; **P<0.01; ***P<0.001; two-

tailed Student’s *t* test (n = 6-12).

| **Liver** |  |  |  |
| --- | --- | --- | --- |
| Gene | Control | Meth | P value |
| TNF-α** | 0.44 ± 0.09 | 4.36 ± 0.72 | 0.004 |
| Interleukin-6 * | 0.02 ± 0.01 | 0.13 ± 0.03 | 0.044 |

| **Heart** |  |  |  |
| --- | --- | --- | --- |
| Gene | Control | Meth | P value |
| TNF-α *** | 0.69 ± 0.06 | 2.07 ± 0.22 | 0.0004 |
| Interleukin-6 * | 0.007 ± 0.001 | 0.02 ± 0.005 | 0.015 |

| **Skin** |  |  |  |
| --- | --- | --- | --- |
| Gene | Control | Meth | P value |
| TNF-α* | 0.37 ± 0.17 | 1.15 ± 0.18 | 0.015 |
| Interleukin-6 ** | 0.05 ± 0.02 | 0.24 ± 0.045 | 0.006 |
