# Supplementary material for: Methamphetamine Accelerates Cellular Senescence through Stimulation of De Novo Ceramide Biosynthesis
Source: PLoS One. 2015 Feb 11;10(2):e0116961. doi: 10.1371/journal.pone.0116961 (PMC4324822; doi:10.1371/journal.pone.0116961)
Supplement: S8 Table — (DOCX) [file pone.0116961.s019.docx]

**Table S8:** Sequences of RT-PCR primers utilized in the present study.

**Mouse primers**

**Transcript Sequence**____________________________

Lass 1 F: 5’-CGTAAGGACTCGGTGGTCAT-3’

R: 5’-CTGACGTCATGCAGGAAGAA-3’

Lass 2 F: 5’-GAAGCCAGCTGGAGATTCAC-3’

R: 5’-GACATCAGAGGCAATGCTGA-3’

Lass 3 F: 5’-CTCTGGGAGGTTTGGAATGA-3’

R: 5’-CAGGTGGTGGATGACATGAG-3’

Lass 4 F: 5’-CCTGCTGGAGGTTTGTCTTC-3’

R: 5’-GGCAAAGTGATCAGCAGTGA-3’

Lass 5 F: 5’-ATTTATTGCCAAGCCCTGTG-3’

R: 5’-AACCAGCATTGGATTTTTCG-3’

Lass 6 F: 5’-CTGAAGAACACGGAGGAAGC-3’

R: 5’-TATGGCACATGGTTTGGCTA-3’

Sptlc1 F: 5’-AGTGGTGGGAGAGTCCCTTT-3’

R: 5’-CAGTGACCACAACCCTGATG-3’

Sptlc2 F: 5’-CCTGTCAGCAGCTCATACCA-3’

R: 5’-CACACTGTCCTGGGAGGAAT-3’

TNF-α F: 5’-AGCCCCCAGTCTGTATCCTT-3’

R: 5’-CTCCCTTTGCAGAACTCAGG-3’

IGF-1 F: 5’-CTACCAAAATGACCGCACCT-3’

R: 5’-CACGAACTGAAGAGCATCCA-3’

β actin F: 5’-AGCCATGTACGTAGCCATCC-3’

R: 5’-CTCTCAGCTGTGGTGGTGAA-3’

18S F: 5’-CGCGGTTCTATTTTGTTGGT-3’

R: 5’-AGTCGGCATCGTTTATGGTC-3’

NF-κB F: 5’-CTGACCTGAGCCTTCTGGAC-3’

R: 5’-GCAGGCTATTGCTCATCACA-3’

IL-1β F: 5’-GCCCATCCTCTGTGACTCAT-3’

` R: 5’-AGGCCACAGGTATTTTGTCG-3’

P21 F: 5’-GCCTTAGCCCTCACTCTGTG-3’

R: 5’-AGGGCCCTACCGTCCTACTA-3’

P53 F: 5’-AGAGACCGCCGTACAGAAGA-3’

R: 5-CTGTAGCATGGGCATCCTTT-3’

P16 F: 5’- CCCGCCTTTTTCTTCTTAGC-3’

R: 5’- TTCTCATGCCATTCCTTTCC-3’

P15 F: 5’- GGTGGGTGCAGTCAGTACCT-3’

F: 5’- CGAGCTGGAGGTGACTTCTC-3’

**Rat primers**

**Transcript Sequence**_______________________________

β actin F: 5’-AGCCATGTACGTAGCCATCC-3’

R: 5’-CTCTCAGCTGTGGTGGTGAA-3’

P21 F: 5’-TCAGTGGACCAGAAGGGAAC-3’

R: 5’-GGTCCCCATCCCAGATAAGT-3’

P53 F: 5’-GTCTACGTCCCGCCATAAAA-3’

R: 5’-AGGCAGTGAAGGGACTAGCA-3’

P16 F:5’-GTGCGGTATTTGCGGTATCT-3’
 R: 5’-CCAGAAGTGAAGCCAAGGAG-3’

P15 F: 5’-TCACCAGACCTGTGCATGAT-3’

R: 5’-AGATAGGGCTGGGGAGAAAA-3’
